# Symmetry Breaking Induced by Chiral Phosphonic Acids in a 2D Tin-Halide Perovskite

**DOI:** 10.1021/jacs.5c11860

**Published:** 2025-09-29

**Authors:** Margherita Taddei, Junxiang Zhang, Md Azimul Haque, Colin McLeod, Steven P. Harvey, Yifan Dong, Laura T. Schelhas, Stephen Barlow, Jeffrey L. Blackburn, Joseph M. Luther, Seth R. Marder, Matthew C. Beard

**Affiliations:** † 53405 National Renewable Energy Laboratory, Golden, Colorado 80401, United States; ‡ Renewable and Sustainable Energy Institute, University of Colorado Boulder, Boulder, Colorado 80309-0027, United States; § Materials Science and Engineering Program, University of Colorado Boulder, Boulder, Colorado 80309-0027, United States; ∥ Department of Chemical and Biological Engineering and Department of Chemistry, University of Colorado Boulder, Boulder, Colorado 80309-0027, United States

## Abstract

The ability to induce and modulate chirality in metal halide perovskite semiconductors (MHPs) using chiral additives expands the compositional design space and offers a means of tuning chiroptical properties. Motivated by the ability of phosphonic acids to interact with metal ions, we designed three chiral phosphonic acids (CPAs) to impose chirality in otherwise achiral 2D MHP, phenylammonium tin iodide (PA_2_SnI_4_). We found that both the position of the phosphonic acid relative to the bond between the two naphthalene rings (i.e., the chiral axis) and the distance between the phosphonic acid and the binaphthyl chiral units significantly impact the transfer of structural chirality into the MHP lattice. The compound with a phosphonic acid directly bound to one of the naphthalene rings at the carbon adjacent to the chiral axis resulted in the largest circular dichroism dissymmetry factor of the three phosphonic acids. Furthermore, optical pump-terahertz probe measurements reveal an increase in the charge carrier mobility in the MHPs following the addition of CPAs. This dual functionality of CPAs in inducing chirality and improving charge transport properties in MHPs is promising for chiral-optoelectronic applications.

## Graphical abstract

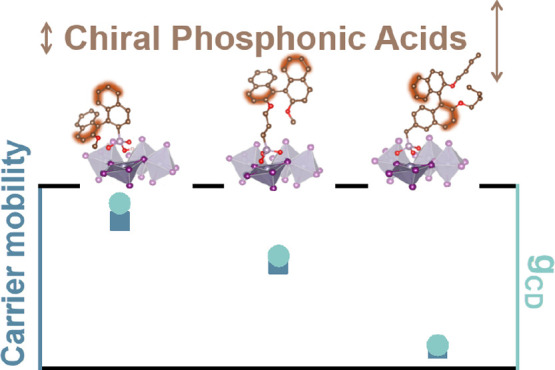

## Introduction

Chirality is a geometric property first discovered on two crystals of sodium ammonium tartrate which were identical chemically but nonsuperimposable-mirror-image versions of each other due to each containing different molecular enantiomer building blocks.^1,2^ Enantiomers show the same optical activity in magnitude but opposite in direction; one enantiomer will differentially absorb left-handed circularly polarized light, while the other will absorb right-handed circularly polarized light.^3,4^ This phenomenon is termed circular dichroism (CD). Since its discovery, chirality has been found across a wide range of molecular systems such as peptides, amino acids, and DNA.^5–7^ Moreover, chirality can be transferred to an achiral inorganic semiconductor through various strategies such as the incorporation of chiral molecules into the crystal lattice,^8,9^ surface functionalization of nanocrystals,^10,11^ and even light-induced,^12^ evaporation processing,^13^ and solvent-driven chirality transfer.^14^

Among promising semiconductors to which chirality can be transferred are metal-halide perovskites (MHPs), a class of hybrid organic–inorganic semiconductors with exceptional optoelectronic properties that have the potential for scalable, low-cost manufacturing in various applications such as photovoltaics,^15^ light-emitting diodes,^16^ thermoelectrics,^17^ and X-ray detectors.^18^ Hybrid MHPs have the generic formula ABX_3_ for a 3D structure and A_2_BX_4_ for a 2D structure, where A is an organic monovalent cation, B is a divalent metal cation, and X a halide anion.^19^ Traditionally, inducing chirality in 2D MHPs has relied on using chiral organic molecules as A-site cations to break the symmetry and impart chiro-optical properties to the inorganic framework.^3,20,21^ However, synthesis of chiral MHPs from achiral building blocks, chiral nucleation, and through chiral additives is emerging as an alternative strategy, further broadening the chemical space for this class of chiral semiconductors.^22–25^ A recent paper reported that a chiral additive can induce remote chirality transfer in achiral 2D MHP polycrystalline films without changing the composition.^26^ In that study, a chiral 1,1′-bi-2-naphthol (BINOL)-derived phosphate compound, (*R*/*S*)-1,1′-binaphthyl-2,2′-diyl-hydrogen-phosphate, exhibited strong chirality transfer in 2D halide perovskite structures, which was attributed to binding to undercoordinated B-site metal cations after deprotonation.

We hypothesize that BINOL-based chiral phosphonic acid (CPA) groups might offer more tunability than BINOL-based chiral phosphates in terms of coordination to the MHP lattice through interaction with the metal cation and/or hydrogen bonding to the halides.^27^ In addition, phosphonic acids have been extensively used in perovskite photovoltaics and light-emitting diodes applications, mostly as metal oxide electrode (FTO/ITO) surface modifiers,^28–30^ but also as additives that self-assemble at the buried interface.^31,32^

Chiral phosphoric acids (CPAs) have been shown to play critical roles as ligands and organo-catalysts for several important chemical synthesis and asymmetric reactions^33^ and have been shown to break symmetry in transition-metal catalysts.^34^ The large and diverse library of CPAs provides a tunable platform for designing chiral MHPs from their achiral MHP counterparts through chirality transfer. In this work, we explore the effectiveness of three different BINOL-derived CPA architectures in transferring chirality to an achiral 2D MHP. The CPAs break the structural symmetry of the achiral MHP, specifically by removing mirror planes perpendicular to the inorganic layers, thereby generating a chiral MHP. Importantly, both the position of attachment of the phosphonic acid relative to the carbon–carbon bond connecting the two naphthalene rings, i.e., the chiral axis, and the distance between the phosphonic acid group and the BINOL chiral group play significant roles in determining the magnitude of the chirality transfer to the MHP as evidenced by both CD and optical pump terahertz probe (OPTP) spectroscopy, suggesting design principles for chirality transfer.

## Results and Discussion

In this work, chirality transfer from a chiral component to a proximal achiral target can be attributed to an asymmetric distortion of the MHP lattice induced by the interactions with the chiral CPA (Figure 1a). We performed FTIR measurements to investigate the chemical interaction between the CPA and the perovskite lattice (Figures S1, S2, and Supporting Information Note S1).^35,36^ In Figure S1b, we found evidence that CPA is deprotonated when added to the PA_2_SnI_4_ perovskite and that these molecules preferentially attach to the perovskite through these P–O bonds rather than bonding (e.g., through H-bonding) to other CPA molecules. The presence of P=O stretching modes in the PA_2_SnI_4_/CPA spectrum confirms the incorporation of CPA into the film, and the insensitivity of these modes to perovskite binding suggests that the P=O bonds are not significantly perturbed in PA_2_SnI_4_/CPA (Figure S1c). Depending on the CPA handedness, the MHP lattice distorts thereby generating left or right-handed structures,^37^ characterized by the opposite sign of the CD spectra (Figure 1b).

**Figure 1:**
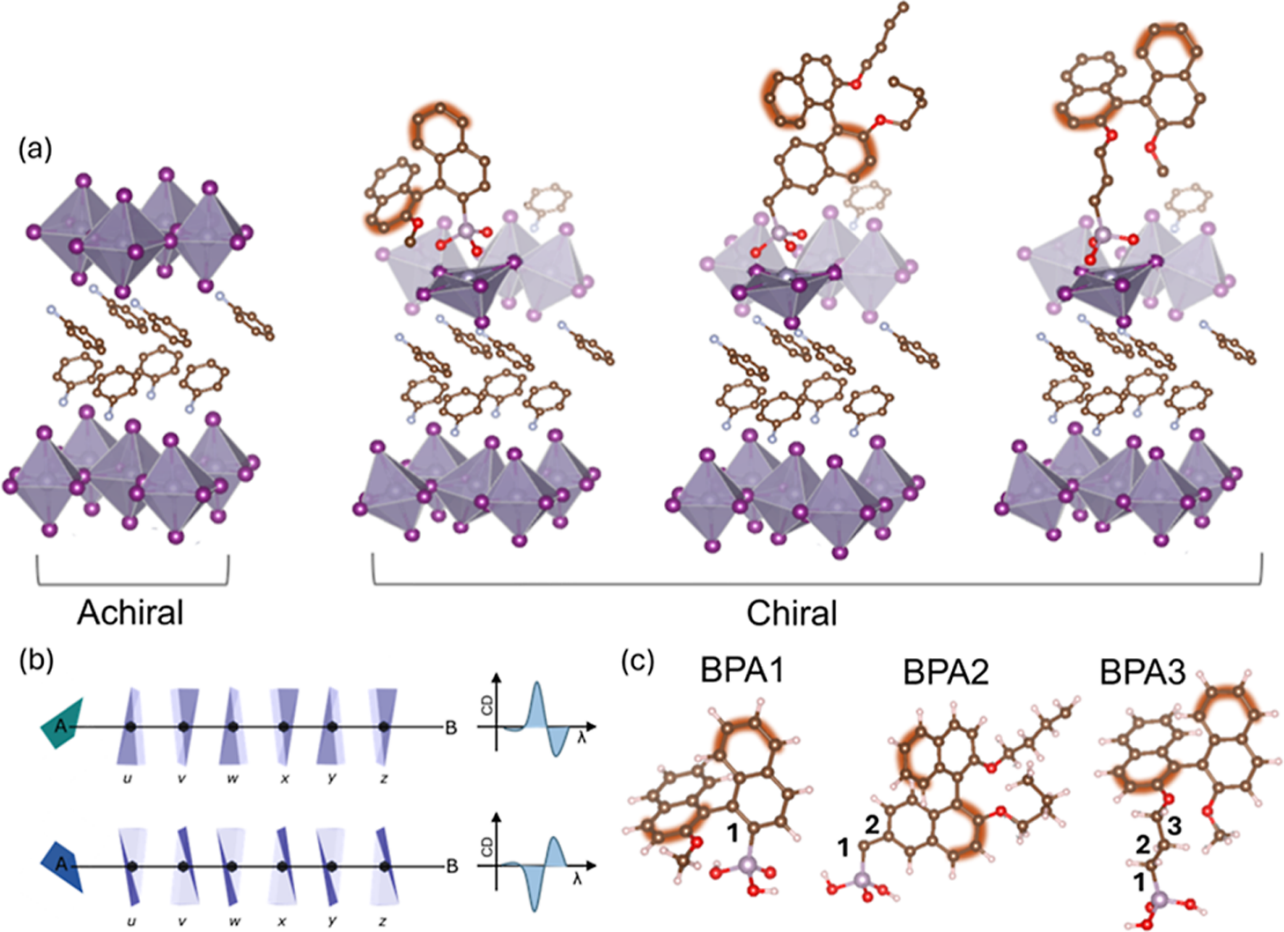
(a) Schematic visualization of the structural chiral distortion after the addition of the three CPAs, assuming that they substitute missing terminal halide ions. The SnI_6_ octahedra are shaded in gray, the iodide ions are the purple vertex of the octahedra, and the phenylammonium cations are positioned in between the octahedra. Carbon atoms are shown in brown, oxygen in red, and phosphorus in gray. (b) Scheme of chirality transfer effect via chiral additive addition, where u, v, w, x, y, and z represent distinct layers in the perovskite with each subsequent increasingly further from the chiral ligand labeled A. (c) 3D rendering of the 3 CPAs used (only the acidic hydrogen atoms are shown for clarity).

To understand how the chirality transfer from BINOL-derived CPAs to the achiral MHP PA_2_SnI_4_ (PA = phenylammonium, i.e., anilinium) is affected by the phosphonic acid substitution position on the binaphthol ring and distance from the chiral axis, we synthesized a series of chiral binaphthalene phosphonic acids (Figure S3): (1*R*)-(2′-methoxy­[1,1′-binaphthalen]-2-yl)­phosphonic acid (*R*-BPA1), (1*S*)-(2′-methoxy-[1,1′-binaphthalen]-2-yl)­phosphonic acid (*S*-BPA1), (1*R*)-((2,2′-dibutyloxy­[1,1′-binaphthalen]-6-yl)­methyl)­phosphonic acid (BPA2), and (1*R*)-(3-((2′-methoxy­[1,1′-binaphthalen]-2-yl)­oxy)­propyl)­phosphonic acid (BPA3). The chemical structures of each CPA are shown in Figure 1c. The identity and purity of the molecules were determined via ^1^H, ^13^C, and ^31^P NMR spectroscopy, elemental analysis, and mass spectrometry (see Supporting Information Synthetic Methods, Figures S4–S25). Their absorption and CD spectra were collected by dissolving them in isopropanol at a 10^–5^ M concentration (Figures S26 and S27); the CD spectra in each case show a Cotton effect close to the absorption edge consistent with expectations for binaphthyl derivatives, and in the case of BPA1, a sign inversion is observed between the two enantiomers.

To study the chirality transfer effect, various molar equivalents of CPAs (Figure 2a) for each Sn^2+^ ion were introduced into the PA_2_SnI_4_ perovskite precursor solution, and the solutions were subsequently spin-coated onto glass substrates. PA_2_SnI_4_ was selected as the host as this composition has been reported to give the highest CD dissymmetry factors (*g*_CD_) in a previous work.^26^ An important advantage of our chirality transfer strategy is the preservation of the MHP composition as the large chiral additives are not able to enter the perovskite lattice. PA_2_SnI_4_ synthesized in the absence of CPAs contains some secondary phases, but the formation of these is suppressed after addition of CPAs (Figure S28). We monitored the XRD patterns over a 3 h period and observed no new phases for pristine PA_2_SnI_4_ and PA_2_SnI_4_/BPA1 films while there was a reduction in the crystallinity (Figures S29 and S30). Addition of the CPAs induces a CD signal at the wavelengths near the band edge of PA_2_SnI_4_, confirming the successful chirality transfer from the CPAs to the MHP lattice (Figure 2b,c). To quantify the chiroptical activity, *g*_CD_ was compared between the three CPAs (Figure 2d). *g*_CD_ was calculated using CD and absorption using eq 1.

1 $$g_{CD}=\frac{CD\left(\lambda \right)}{32,980\times Abs\left(\lambda \right)}$$

**Figure 2:**
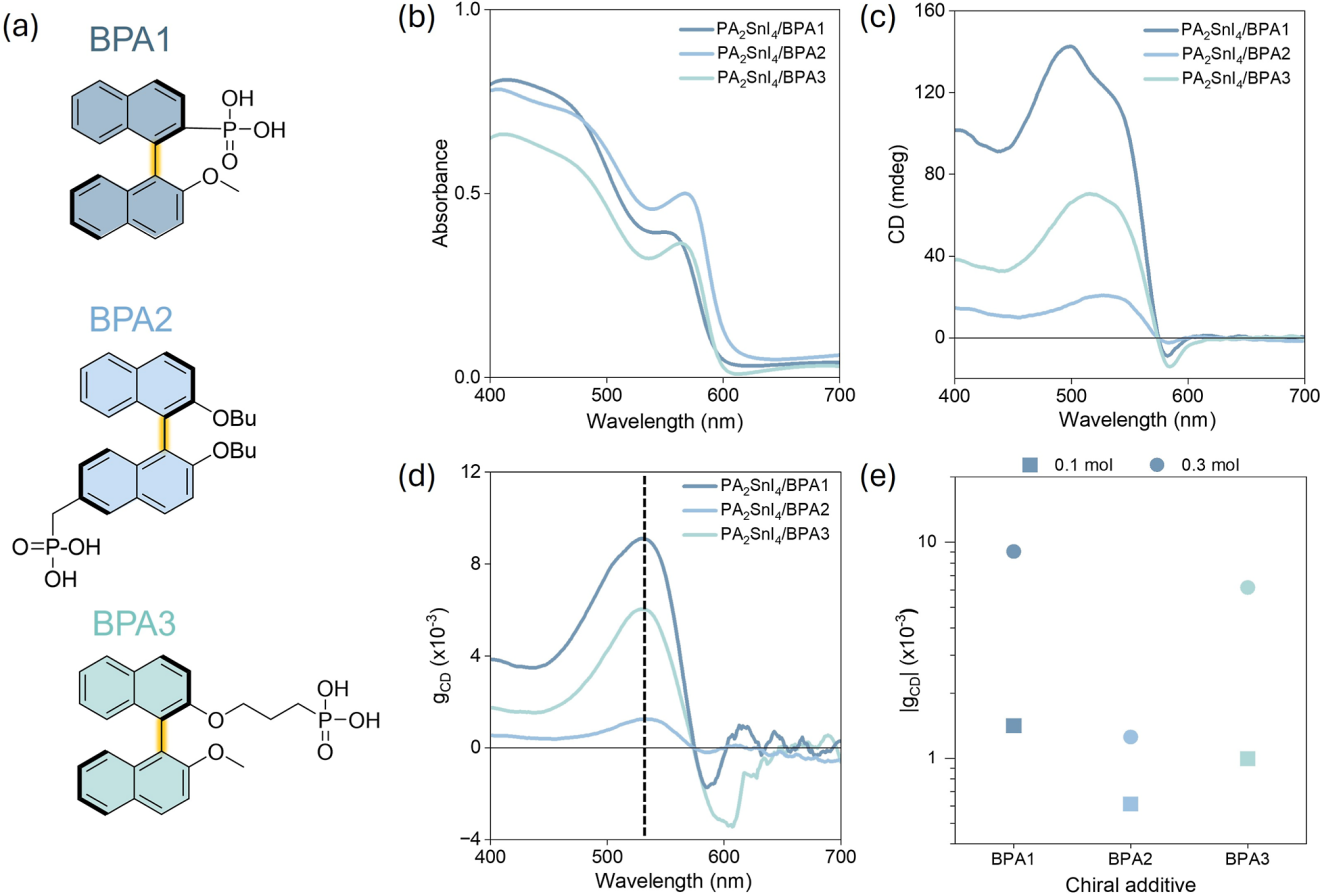
(a) Chemical structure of (1*R*)-(2′-methoxy­[1,1′-binaphthalen]-2-yl)­phosphonic acid (BPA1), (1*R*)-((2,2′-dibutyloxy­[1,1′-binaphthalen]-6-yl)­methyl)­phosphonic acid (BPA2), (1*R*)-(3-((2′-methoxy­[1,1′-binaphthalen]-2-yl)­oxy)­propyl)­phosphonic acid (BPA3). (b) UV–vis spectra of PA_2_SnI_4_ perovskite films with addition of 0.3 mol equiv of BPA1 (dark blue), BPA2 (blue), and BPA3 (green). (c) CD spectra and (d) *g*_CD_ values calculated using eq 1 for the same samples as shown in (a), the wavelength (531 nm) at which the *g*_CD_ is reported is shown with a dashed line. (e) Absolute values of *g*_CD_ at 531 nm for samples with addition of 0.1 (square) and 0.3 (circle) molar equivalents of BPA1, 2, and 3.

PA_2_SnI_4_/BPA1 shows the highest *g*_CD_ values of the three CPAs for the cases of both 0.1 and 0.3 mol equiv; this is presumably due to the shorter distance between the phosphonic acid group and the chiral axis (Figures 2e and S31). PA_2_SnI_4_/BPA2 shows the smallest *g*_CD_ values: although the bridge between the phosphonic acid group and the binaphthalene moiety in BPA2 is shorter than that for BPA3, it is farther from the carbon–carbon bond connecting the two naphthalene groups (i.e., chiral axis), and as such, only one naphthalene ring might closely interact with the Sn halide lattice. We examined the extent to which the diastereomeric ethyl methylene and methyl resonances of the diethyl phosphonate precursors to BPA1, 2, and 3 (Figure S32 and Supporting Information Note S2) were distinguishable by ^13^C­{^1^H} NMR as an indicator of the extent to which the phosphonic acid/phosphonate groups in these systems “feel” the impact of the chiral BINOL group. The CH_2_ chemical shift differences for methylene and methyl resonances for the precursor of BPA1 is much larger than those of the BPA2 and BPA3 precursors, following the trend BPA1 > BPA2 > BPA3, while the CD of the films follows the order PA_2_SnI_4_/BPA1 > PA_2_SnI_4_/BPA3 > PA_2_SnI_4_/BPA2. This difference may reflect different steric effects on the surface coverage and/or the mode of binding of phosphonic acid to the perovskite. Additionally, it is worth noting that the crystallinity of PA_2_SnI_4_/BPA2 and PA_2_SnI_4_/BPA3 is significantly reduced compared to that of PA_2_SnI_4_/BPA1 (Figure S33). A similar trend emerged when adding 0.1 mol equivalent of CPAs in a lead-based composition PA_2_PbI_4_ (Figure S34).

Next, we focus on the concentration dependence of BPA1 as it shows the highest chirality transfer to the MHP by adding different amounts of both *R*- and *S*-enantiomers of BPA1. Interestingly, the CD spectra shape changes with increasing amounts of BPA1, suggesting a different degree of interactions occurring between the chiral molecule and MHP (Figures 3a–d and S35–S37). Of the three concentrations examined, the highest value is reached for 0.3 mol equiv (or 50 mg/mL) (Figure 3b). At the higher concentration of 0.6 mol equiv, the CD peak at ca. 500–530 nm changes sign for both the *R* and *S* enantiomers (Figure 3c). The inversion of CD sign might be attributable to changes in the perovskite crystallization and the dihedral angle between the naphthyl rings under high concentrations.^38,39^ A maximum *g*_CD_ of 1.5 × 10^–2^ is achieved for PA_2_SnI_4_/BPA1, which surpasses most of the structurally chiral MHPs based on chiral ammonium cations.^40^ In addition, there is a nonlinear increase in *g*_CD_ with the increasing concentration of BPA1 (Figure 3d). Linear dichroism and linear birefringence (LD·LB) effects, intrinsic to polycrystalline thin films, have recently been recognized as legitimate chiroptical processes rather than optical artifacts.^41,42^ In our study, we demonstrate that the CD signal arises only with the addition of a chiral additive and, despite some LD·LB effects, no full sign flip occurs when measuring the back side, distinguishing it from structurally chiral perovskites (Figure S38).^4,43^

**Figure 3:**
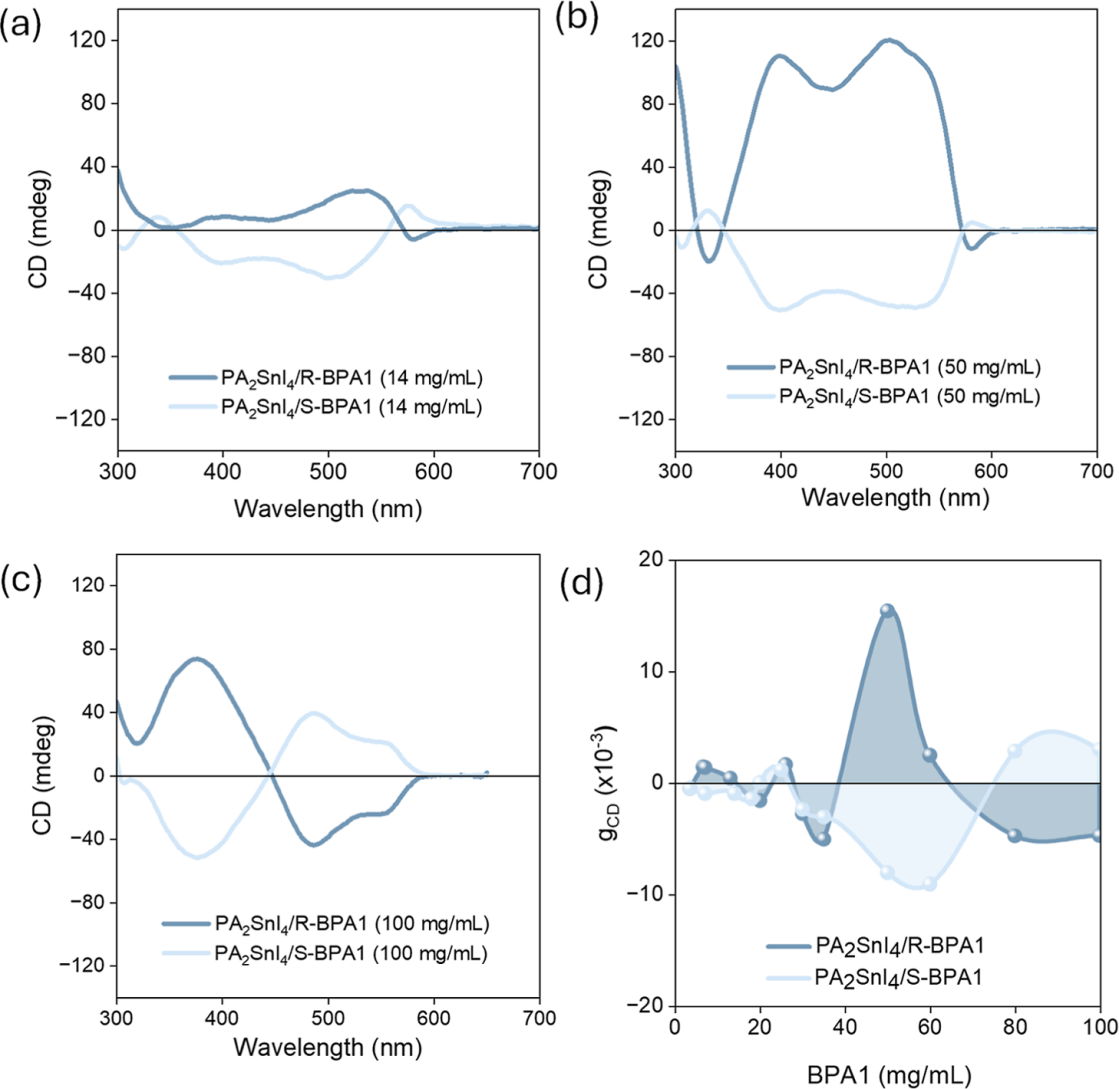
(a) CD spectra of films with addition of *R*-/*S*-BPA1 at low concentration of 14 mg/mL (0.1 mol equiv), (b) 50 mg/mL (0.3 mol equiv), (c) 100 mg/mL (0.6 mol equiv), and (d) *g*_CD_ values at 540 nm of perovskite films as a function of *R*- and *S*-BPA1 addition in the perovskite precursor solution.

Addition of BPAs can also affect the microstructure of the MHP films. Scanning electron microscopy (SEM) images of PA_2_SnI_4_ after addition of CPAs show an improvement in the film quality compared to pristine PA_2_SnI_4_ (Figures 4a–c, S39 and S40). PA_2_SnI_4_/BPA1 forms smoother films with fewer pinholes compared to those of pristine PA_2_SnI_4_, PA_2_SnI_4_/BPA2, or PA_2_SnI_4_/BPA3. The film morphology is already smoother for low concentrations of BPA1, resulting in a more compact and uniform film (Figure S41). Time-of-flight secondary ion mass spectrometry (ToF-SIMS) was performed to visualize the distribution of the CPAs in the MHP film. For the concentrations producing the highest *g*_CD_ values, all CPAs show a large presence at the buried interface of the MHP film (Figures 4d–f, S42 and S43). This accumulation at the perovskite–substrate interface suggests that the migration of the molecule during film crystallization follows a top-down crystallization process.^31,44,45^ In PA_2_SnI_4_/BPA2, we also found a significant amount of the CPA on the top surface, likely accumulating during crystallization due to its larger size compared to the other CPAs.

**Figure 4:**
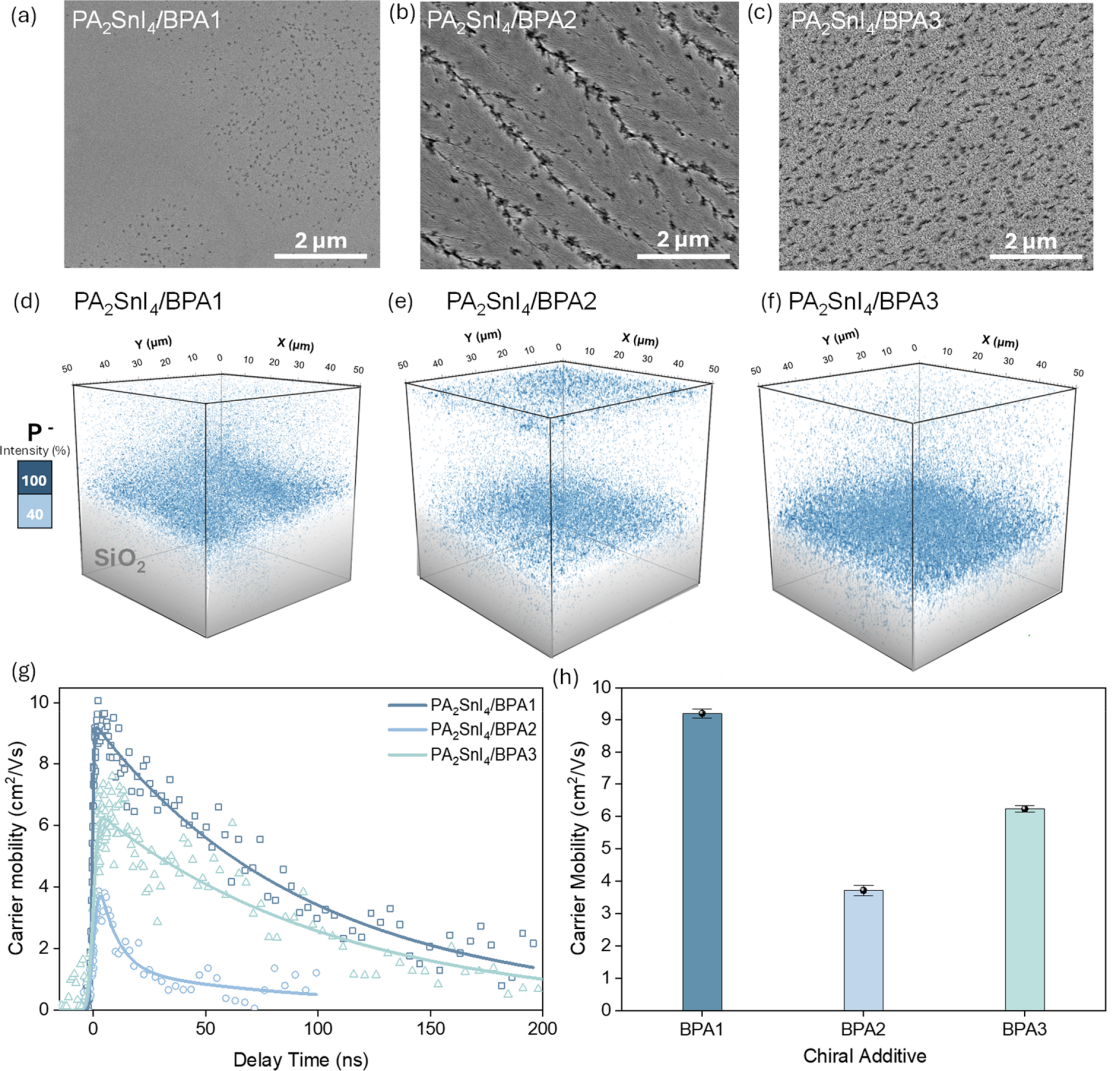
(a) SEM images of PA_2_SnI_4_ films with addition of 0.3 mol equiv of BPA1, (b) BPA2, and (c) BPA3. (d–f) ToF-SIMS 3D distribution of P^–^ (*m*/*z* = 30.9679) fragments with minimum normalized intensity of 40% in clear blue and maximum of 100% in dark blue. (g) OPTP measurement of the PA_2_SnI_4_ films with addition of 0.3 mol equiv of BPA1, BPA2, and BPA3 exciting with 500 nm laser at 7 mW/cm^2^ for PA_2_SnI_4_/BPA1 and PA_2_SnI_4_/BPA2 and 8.8 mW/cm^2^ for PA_2_SnI_4_/BPA3. (h) Carrier mobility values extracted from fitting of OPTP measurements.

To characterize how the addition of the CPAs impacts the charge-carrier transport, we employed OPTP spectroscopy. In these measurements, we monitored the change in the transmittance of the terahertz pulse through the MHP samples before (Figure S44) and after photoexcitation.^46–48^ Figure 4g shows the carrier mobility decay over time, extracted using eq 2 and the procedure described in the Experimental Methods section. In Figure 4h, we show that PA_2_SnI_4_/BPA1 has a higher in-plane mobility (9.19 ± 0.14 cm^2^ V^–1^ s^–1^) than PA_2_SnI_4_/BPA2 (3.71 ± 0.14 cm^2^ V^–1^ s^–1^) and PA_2_SnI_4_/BPA3 (6.30 ± 0.10 cm^2^ V^–1^ s^–1^), which can be attributed to its higher crystallinity. The mobility of the control film (3.72 ± 0.16 cm^2^ V^-1^ s^-1^) is shown in Figure S45. Notably, the carrier mobility follows the same trend as the chiroptical activity (*g*_CD_), suggesting some correlation (Figure S46) that warrants further investigation.

## Conclusions

In this work, we demonstrated that employing BPA1, where the phosphonic acid is connected directly to a naphthalene ring at a site ortho to the carbon–carbon bond connecting the two naphthalene rings (i.e., the chiral axis), as an additive, enables significantly more effective chirality transfer in 2D halide perovskites, compared to phosphonic acids BPA2 and BPA3. In addition, BPA1 led to improved morphology, crystallinity, and higher CD and charge carrier mobility. These results highlight the critical role of molecular structure, particularly chain length, steric hindrance, and distance from the chiral center, in determining the efficacy of chirality transfer and charge carrier dynamics. These newly developed CPAs may also be used as surface modifiers, offering potential for interface engineering in chiral opto-spintronic devices.

## Experimental Methods

Phenylammonium iodide (PAI) was purchased from GreatCell Solar. Anhydrous dimethylformamide (DMF) and SnI_2_ AnhydroBeads, −10 mesh, 99.99% trace metal basis were purchased from Sigma-Aldrich. *P*-[(1*R*)-2′-Methoxy­[1,1′-binaphthalen]-2-yl]­phosphonic acid (BPA1), *P*-(1*R*)-2,2′-bis­(butyloxy)-[1,1′-binaphthalene]-6-yl-methylene-phosphonic acid (BPA2), and *P*-[[(1*R*)-2′-methoxy­[1,1′-binaphthalene]-2-yl]­oxy-3,1-propyl]­phosphonic acid (BPA3) synthesis details are listed in the Supporting Information. Diethyl *P*-[(1*R*)-2′-methoxy­[1,1′-binaphthalen]-2-yl]­phosphonate and (*R*)-2-hydroxy-2′-methoxy-1,1′-binaphthyl were synthesized according to the literature.^49,50^ Diethyl *P*-(3-bromopropyl)­phosphonate was purchased from Sigma-Aldrich.

To prepare the metal halide perovskite polycrystalline films, 1 mmol (221 mg) PAI and 0.5 mmol (186 mg) SnI_2_ were dissolved in 1 mL of DMF. The chiral *R*-/*S*-BPA1 molecules were then added to the precursor solution in different quantities from 3.5 to 100 mg/mL, which correspond to 0.025 to 0.3 mol equiv. BPA2 and BPA3 were added with 0.1 and 0.3 mol equiv, which correspond to 20 and 68 mg/mL for BPA2, and 17 and 60 mg/mL for BPA3. The films were then prepared by spin-coating 70 μL of the precursor solution onto a precleaned glass substrate at 4000 rpm for 30 s, followed by an annealing step at 75 °C for 20–60 s depending on when the samples became pink colored. The samples were then sealed in a centrifuge tube under N_2_ for transport, and some were encapsulated using an UV-curable resin.

### Characterization Methods

Ultraviolet (UV)–visible (Vis) absorption spectra were taken on a Cary 4000 spectrometer. CD measurements were acquired by using an Olis DSM 170 spectropolarimeter. X-ray diffraction (XRD) measurements were performed using a Rigaku Ultima IV with Cu Kα radiation at ambient temperature. SEM images were taken on a Hitachi S-4800 scanning electron microscope. ToF-SIMS was done on an ION-TOF TOF-SIMS V spectrometer.

### Optical Pump Terahertz Probe

The fundamental laser pulse with wavelength at 800 nm is generated by a Ti:sapphire amplifier. The pulse repetition rate is 1 kHz. The fundamental pulse is then split into two parts by a beam splitter. One part is sent to an optical parametric amplifier for pump generation at 500 nm. The probe beam had two arms. The first arm generates freely propagating THz pulses that are focused onto a sample, transmitted, and refocused onto a ZnTe detector crystal. The THz pulse overlapped spatially and temporally with the optical pump pulse at the sample position. The delay between the THz pulse and the pump pulse is controlled by a delay stage. The second arm of the probe beam controls a gating near-IR pulse for free space electro-optical sampling of the THz pulses. The gating pulse and transmitted THz pulse are overlapped spatially and temporally at the detector crystal. For the dark scan (*T*), the probe is chopped at a frequency of 500 Hz. For the pump scan (Δ*T*), the pump is then chopped at a frequency of 500 Hz. The average excitation density of the pump was calculated by using a 4 mm pinhole and a Thorlab power meter. To ensure sample stability throughout the 1 h measurement window, during which 10 decay traces were recorded and averaged, the samples were encapsulated inside the glovebox. The MHP samples were deposited on glass and encapsulated with glass using an UV resin inside the N_2_-filled glovebox (O_2_ < 0.1 ppm, H_2_O < 0.1 ppm) just after spin-coating. The carrier mobility value was calculated using eq 2

$${QY}_{cc}\;\mu =-\frac{\varepsilon_{0}\left({2n}_{glass}\right)\;c}{e\;j_{p}\left(1-10^{-OD}\right)\frac{\lambda }{hc}}\frac{\Delta T}{T}$$ 2

where ε_0_ is the vacuum permittivity in C/V m, *n*_glass_ (=1.75) is the refractive index of the glass interfacing the perovskite layer in the front and the back side at the THz frequencies, *c* is the speed of light in m/s, $\frac{\Delta T}{T}$ is the ratio of the photoinduced change in the THz electric field to the transmitted THz electric field in the dark, *e* is the elementary charge in J, OD is the optical density of the perovskite film at the pump wavelength (500 nm) taken from UV–vis, *j*_p_ is the fluence of the pump in J/m^2^, λ is the wavelength of the pump in m, and *h* is the Planck constant in J s. μ is the charge-carrier mobility, and QY_cc_ is the quantum yield of free charges created per photon absorbed. Here, we calculate the QY_cc_ μ product, which is the effective charge-carrier mobility. The curves are then fitted with a triexponential function.

## Supplementary Material



## Notes

FTIR, NMR spectra for all compounds, chiral molecule synthesis details, and additional XRD, SEM, and ToF-SIMS (PDF)
