# Supplementary material for: Symmetry Breaking Induced by Chiral Phosphonic Acids in a 2D Tin-Halide Perovskite
Source: J Am Chem Soc. 2025 Sep 29;147(40):36642–9. doi: 10.1021/jacs.5c11860 (PMC12512100; doi:10.1021/jacs.5c11860)
Supplement: Supplementary file 1 [file ja5c11860_si_001.pdf]

Supporting Information:

Symmetry Breaking Induced by Chiral Phosphonic Acids in a 2D Tin-Halide Perovskite

Margherita Taddei,[1] Junxiang Zhang,[2] Md Azimul Haque,[1,2] Colin McLeod,[2] Steven P. Harvey,[1] Yifan Dong,[1] Laura T. Schelhas,[1,2] Stephen Barlow,[1,2] Jeffrey L. Blackburn,[1] Joseph M. Luther,[1,2] Seth R. Marder,[1,2,3,4]* Matthew C. Beard[1,2]*

[1]National Renewable Energy Laboratory, Golden, CO, 80401, USA
[2]Renewable and Sustainable Energy Institute, University of Colorado Boulder, Boulder, CO, 80309-0027, USA
[3]Materials Science and Engineering Program, University of Colorado Boulder, Boulder, CO, 80309-0027, USA
[4]Department of Chemical and Biological Engineering and Department of Chemistry, University of Colorado Boulder, Boulder, CO, 80309-0027, USA
matt.beard@nrel.gov; seth.marder@colorado.edu

Table of Contents

Supplementary Note 1: FTIR Analysis 3

Synthetic Methods 5

NMR Spectra 11

Supporting data part 1: CD, XRD 23

Supplementary Note 2: Impact of Binaphthyl Chirality on the Phosphonic Acid Moiety 26

Supporting data part 2: CD, XRD, SEM, ToF-SIMS, THz 27

References 35

Supplementary Note 1: FTIR Analysis

We employed diffuse reflectance infrared Fourier Transform spectroscopy (DRIFTS) to explore the binding mode of the chiral phosphonic acids (using (1*R*)-(2′-methoxy[1,1′-binaphthalen]-2-yl)phosphonic acid**,** named BPA1 hereafter, as an example) to the 2D perovskite. **Figure S1a** displays the broad survey spectra for BPA1, highlighting some key regions of interest. The region from ca. 800 – 1100 cm^{-1} (Region 1) contains peaks corresponding to OH and P-O vibrational modes of the phosphonic acid moiety, while the region from 1150 – 1400 cm^{-1} (Region 2) contains peaks corresponding primarily to P=O vibrational modes. Additional OH modes can be observed in the regions of ca. 1600 cm^{-1} (Region 3), corresponding to OH deformations, and 2200 cm^{-1} (Region 4), corresponding to OH stretching in P-OH groups involved in H-bonding.

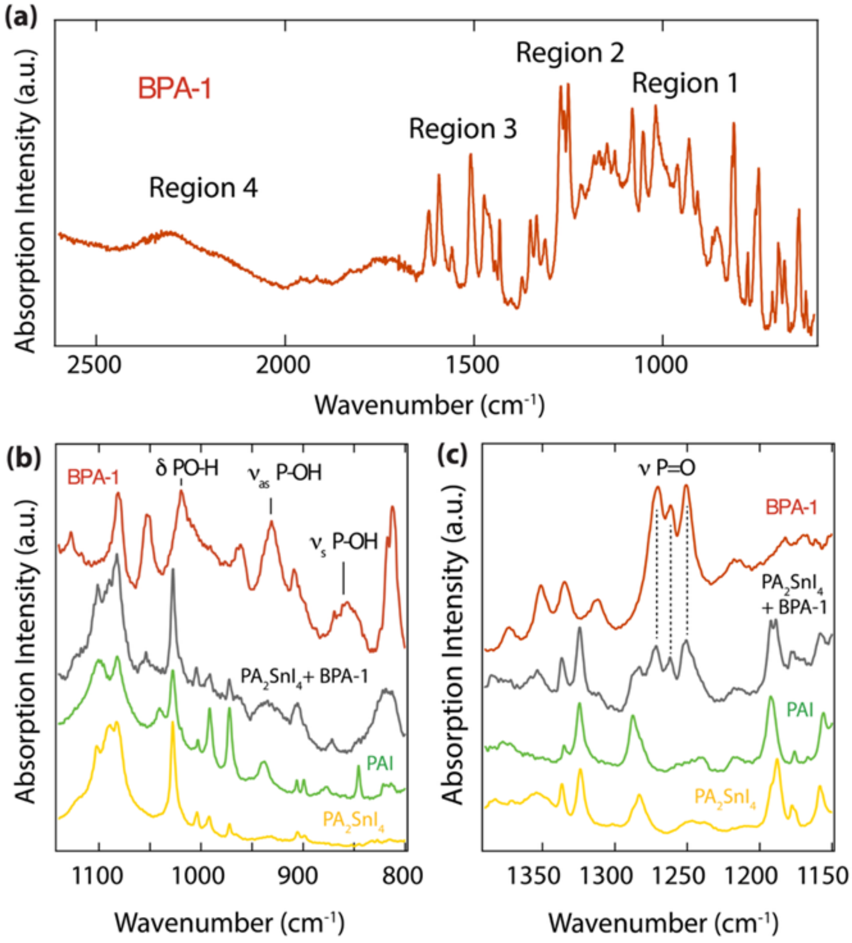

Figure S1: **a)** Survey DRIFTS spectrum of BPA1, highlighting several regions of interest discussed more in the text. **b)** Zoom of Region 1, highlighting symmetric (ν_s P-OH) and anti-symmetric stretching (ν_{as} P-OH) modes of P-OH bonds and bending modes (δ PO-H) of PO-H bonds that disappear when BPA1 is bonded to PA_2SnI_4. **c)** Zoom of Region 2, highlighting the insensitivity of P=O stretching (ν P=O) modes when BPA1 is bonded to PA_2SnI_4.

Figures S1b and **S1c** show zoomed in versions of Regions 1 and 2, while **Figure S2a – S2c** show zoomed versions of Regions 3 – 4 and the fingerprint region (400 – 800 cm^{-1}). To disentangle the

contributions from the phenylammonium A-site cation and the CPA, we measured the DRIFTS spectra for each of these molecules, in addition to the PA_2SnI_4 both with and without BPA1. In all regions, DRIFTS peaks found in the PA_2SnI_4 2D perovskite spectrum (yellow trace) correspond solely to the phenylammonium A-site cation and correlate directly to the vibrational modes observed for PAI (green trace). Minor variations in relative peak intensities and energies result from the incorporation of PA into the 2D perovskite framework. Similarly, the incorporation of BPA1 into the 2D perovskite results in the appearance of most predominant BPA1 peaks (red trace) into the composite spectrum (PA_2SnI_4 + BPA1, black trace). However, the disappearance or shifting of several important peaks can reveal key features of the bonding details between the CPA and perovskite.

In Region 1, three peaks in the BPA1 spectrum correspond to characteristic modes of the P-OH phosphonic acid protons. The 860 cm^{-1} and 930 cm^{-1} peaks correspond to symmetric stretching (ν_s P-OH) and anti-symmetric stretching (ν_{as} P-OH), respectively, and the 1020 cm^{-1} peak corresponds to PO-H bending (δ PO-H). Importantly, these peaks are absent in the composite spectrum, signifying deprotonation of the phosphonic acid protons when BPA1 is added to the 2D perovskite. This conclusion is also supported by a loss of other peaks reported to reflect the acidic protons – namely peaks around 1593 cm^{-1} and 1620 cm^{-1}, previously assigned to OH deformation and the broad peak envelope in the range of 2200 – 2400 cm^{-1} previously assigned to OH stretching (Regions 3 – 4, **Figure S2a – S2b**). Both of these regions have been noted to be characteristic of phenyl phosphonic acid molecules that are agglomerated by hydrogen bonding. These changes all point to a mechanism whereby the BPA1 molecules are deprotonated upon bonding to the 2D perovskite. They also suggest that, regardless of any molecular clustering at perovskite surfaces or grain boundaries, the P-O moieties bond predominantly with the perovskite and not to other BPA1 molecules (i.e. via H-bonding). This observation is similar to the deprotonation previously observed for binaphthol phosphate (BHP) chiral modifiers upon bonding to 2D perovskites.[1]

We turn to Region 2 to assess the possibility that the P=O bond is also involved in bonding between the BPA1 and the perovskite. The grouping of peaks between ca. 1250 – 1313 cm^{-1} is in the characteristic range expected for the P=O stretching mode (ν P=O). Interestingly, this peak envelope does not change when BPA1 is incorporated into the 2D perovskite. All peak positions, and even the relative peak intensities, are essentially identical for the free BPA1 molecule and the composite spectrum of PA_2SnI_4/BPA1. This result suggests that the P=O of the phosphonic acid moiety remains intact upon BPA1 binding to the perovskite, implying that the bonding of the phosphonic acid with the perovskite is most likely bidentate, i.e. forming the surface-bound phosphonate. Interestingly, this observation contrasts with the appreciable shift of P=O modes for BHP *phosphate* modifiers to 2D perovskites.[1]

In theory, the bidentate bonding of BPA1 to the 2D perovskite to form the surface-bound phosphonate should result in the formation of two new peaks corresponding to the symmetric and anti-symmetric stretching of the phosphonate P-O bonds. Peaks for these modes have been shown to occur at

ca. 1060 cm^{-1} and 1080 cm^{-1} (Region 1, **Figure S1b**). While we do observe peaks at 1053 cm^{-1} and 1083 cm^{-1}, these peaks are also present in the neat BPA1 spectrum, and the 1083 cm^{-1} peak is also coincident with a peak observed for the phenylammonium A-site cation. Thus, we cannot point definitively to these peaks as evidence for the bidentate BPA1 binding. The DRIFTS measurement is also unable to identify any potential bonding between Sn and the O atoms of the BPA1. The oxidation state of tin in the perovskite is expected to be Sn(II). Sn(II) binding to O, e.g. in SnO, has a characteristic IR-active mode at 260 cm^{-1}, well below the lower energy limit of the DRIFTS measurement (ca. 400 cm^{-1}). Sn(IV) binding to O, e.g. in SnO_2, has a very strong IR-active mode in the range of ca. 600 – 630 cm^{-1}, which can easily be resolved by DRIFTS. We do not observe any modes in this range (fingerprint region, **Figure S2c**), besides peaks that correspond directly to modes of the neat PA and BPA1 molecules, allowing us to rule out any interaction between the inorganic framework and the BPA1 that would change the expected Sn(II) oxidation state to Sn(IV).

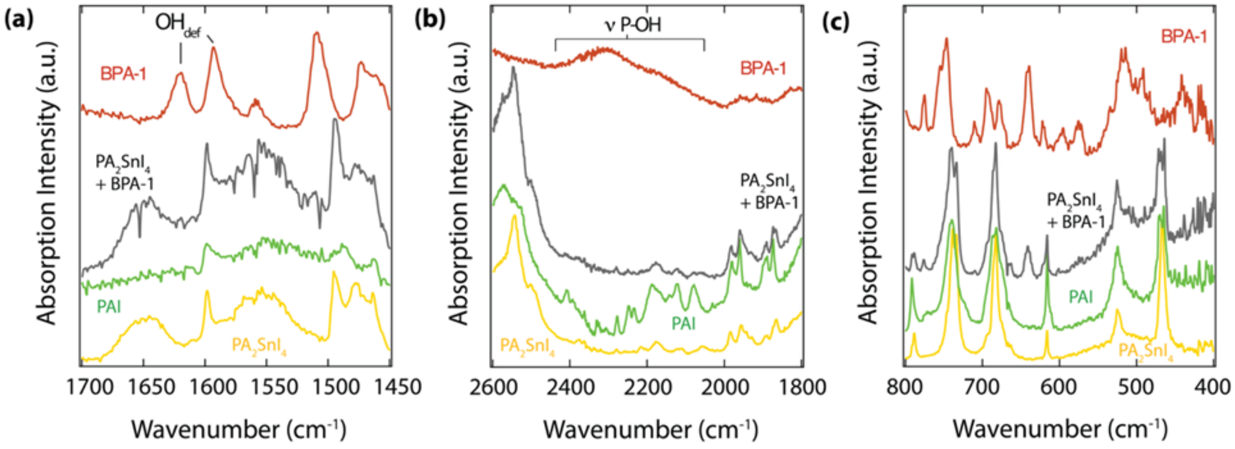

Figure S2: Additional zoomed-in regions of DRIFTS spectra for Region 3 **(a)**, Region 4 **(b)**, and the fingerprint region **(c)**.

Synthetic Methods

1, Diethyl *P*-[(1*R*)-2′-methoxy[1,1′-binaphthalen]-2-yl]phosphonate,[2] 2a, (1*R*)-6-bromo[1,1′-binaphthalene]-2,2′-diol,[3] and 3a, (*R*)-2-hydroxy-2′-methoxy-1,1′-binaphthyl,[4] were synthesized according to literature. Diethyl *P*-(3-bromopropyl)phosphonate was purchased from Sigma-Aldrich.

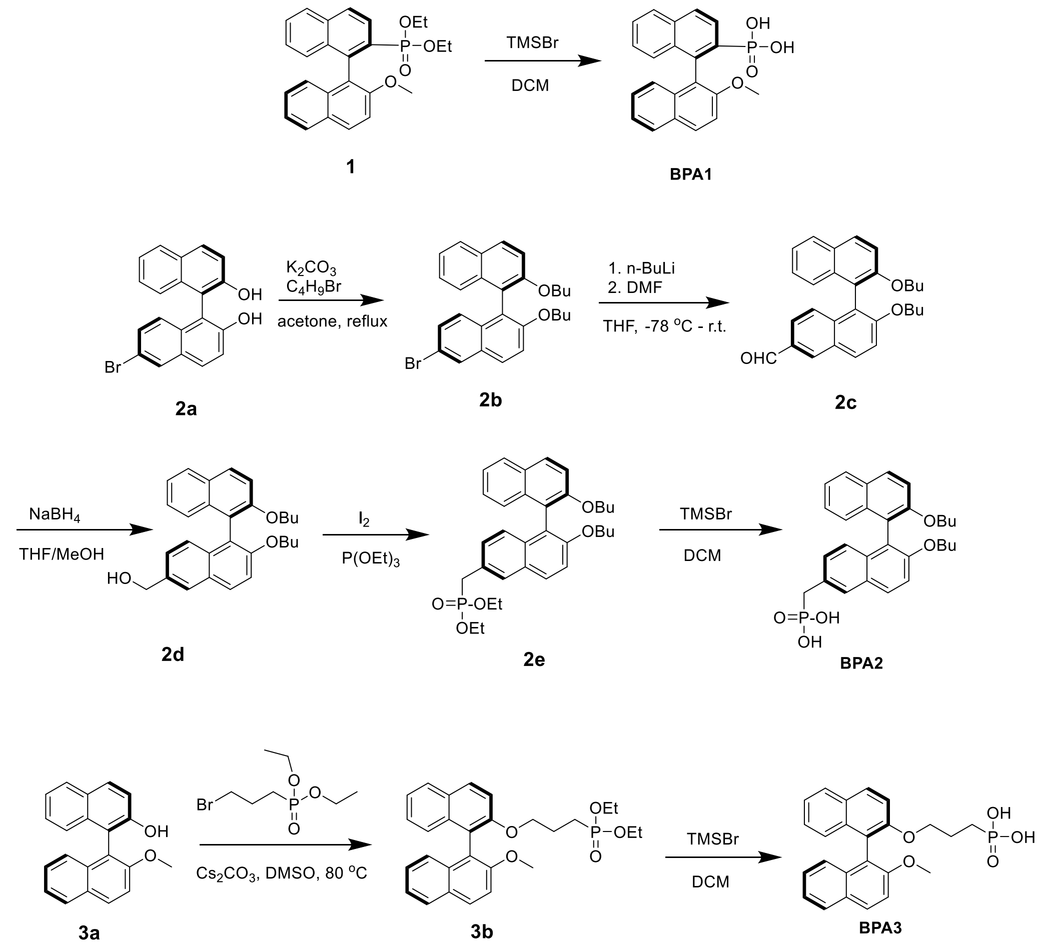

Figure S3: Synthesis of BPA1, BPA2, and BPA3.

Synthesis of *P*-[(1*R*)-2′-methoxy[1,1′-binaphthalen]-2-yl]phosphonic acid (BPA1)

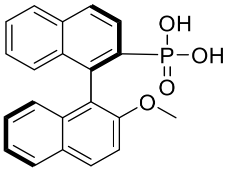

To a dichloromethane (DCM, 20 mL) solution of diethyl *P*-[(1*R*)-2′-methoxy[1,1′-binaphthalen]-2-yl]phosphonate (1, 650 mg, 1.55 mmol) at 0°C was added trimethylsilyl bromide (TMSBr, 0.33 mL, 9 mmol). The resulting mixture was stirred overnight and slowly warmed to room temperature. After TLC indicates the reaction was complete, MeOH (5 mL) and water (30 mL) were added, and the mixture was stirred for 30 min. Volatile solvents were removed by rotary evaporation. The resulting solid was filtered and washed with water and diethyl ether to obtain the desired product (538 mg, 96%) as a white solid. ^{1}H NMR (400 MHz, 0.4 mL DMSO-d_6 and 5 µL D_2O) δ 8.14–7.98 (m, 4H), 7.89 (d, J = 8.1 Hz, 1H), 7.53 (dd, J = 8.7, 6.2 Hz, 2H), 7.31–7.21 (m, 2H), 7.12 (ddd, J = 8.3, 6.7, 1.3 Hz, 1H), 6.92 (d, J = 8.5 Hz, 1H), 6.73 (d, J = 8.5 Hz, 1H), 3.68 (s, 3H). ^{13}C{^{1}H} NMR (101 MHz, 0.4 mL DMSO-d_6 and 5 µL D_2O) δ 155.20, 138.90 (d, J = 9

Hz), 134.57 (d, J = 3 Hz), 134.38, 132.96 (d, J = 15 Hz), 130.85, 129.87, 129.14 (d, J = 10 Hz), 128.66, 128.42, 127.99, 127.61, 127.28 (d, J = 13 Hz), 126.94, 126.76, 126.15, 125.86, 123.29, 121.38 (d, J = 5 Hz), 114.21, 56.35. ^{31}P NMR (162 MHz, 0.4 mL DMSO-d_6 and 5 µL D_2O) δ 12.06. HRMS-ESI, calcd. for $C_{21}H_{16}O_4P$ $(M-H)^-$: 363.0792; found 363.0795. Note: D_2O is added to simplify the NMR spectra, apparently disrupting hydrogen-bonded species, to which we assign a signal at 6.99 ppm in the ^{31}P NMR spectra in anhydrous DMSO-d_6 (See Figure S4).

Synthesis of (1*R*)-6-bromo-2,2′-bis(butyloxy)-1,1′-binaphthalene (2b)

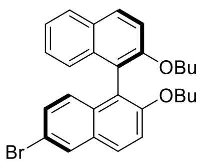

To a 250 mL 2-neck round bottom flask at room temperature, (1*R*)-6-bromo[1,1′-binaphthalene]-2,2′-diol (2a, 5.0 g, 13.4 mmol), K_2CO_3 (7.4 g, 53.2 mmol, 4 equiv), and acetone (60 mL) were added. While stirring, 1-bromobutane (11.0 g, 80.5 mmol, 6 equiv) was added and the mixture was heated at reflux for 15 h. The resulting product was yellow and contained two distinct precipitates. The mixture was filtered to isolate the liquid product and washed using ethyl acetate. The crude was purified by silica gel chromatography (4:1 hexanes/DCM) to elude product as a yellow oil (2.3 g, 37%). ^1H NMR (400 MHz, $CDCl_3$) δ 8.02 (d, J = 2.0 Hz, 1H), 7.95 (d, J = 9.0 Hz, 1H), 7.86 (t, J = 8.4 Hz, 2H), 7.43 (t, J = 8.5 Hz, 2H), 7.33 (t, J = 7.5 Hz, 1H), 7.27–7.18 (m, 2H), 7.11 (d, J = 8.5 Hz, 1H), 7.04 (d, J = 9.0 Hz, 1H), 4.11–3.90 (m, 4H), 1.49–1.33 (m, 4H), 1.08–0.95 (m, 4H), 0.74–0.62 (m, 6H). $^{13}C\{^1H\}$ NMR (101 MHz, $CDCl_3$) δ 154.85, 154.51, 134.09, 132.78, 130.26, 129.68, 129.30, 129.26, 129.19, 128.11, 127.85, 127.45, 126.19, 125.23, 123.45, 120.91, 119.85, 117.14, 116.68, 115.57, 69.32, 69.29, 31.38, 31.33, 18.77, 18.72, 13.58, 13.54. HRMS-ESI, calcd. for $C_{28}H_{29}BrO_2K$ $(M+K)^+$: 515.0983; found 515.0981.

Synthesis of (1*R*)-2,2′-bis(butyloxy)-[1,1′-binaphthalene]-6-carboxaldehyde (2c)

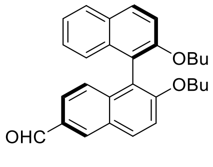

In a 50 mL round bottom flask, under nitrogen, 2b (2.3 g, 5 mmol) was dissolved in anhydrous THF (20 mL). The solution was cooled using a bath of dry ice and isopropanol. n-BuLi (3 mL, 7.5 mmol, 1.5 equiv) was added dropwise to the solution and stirred for 2 h. Then, 0.77 mL (0.73 g, 10 mmol, 2.0 equiv) of dry DMF was added in drops. This solution was left to stir and come to room temperature overnight. After this, the solution was poured into a mixture of HCl and ice water. The resulting mixture was extracted three times

with DCM and washed using $NaHCO_3$ aqueous solution. The excess solvent was removed via rotary evaporator and purified using flush column with 3:1 DCM/Hexanes as an eluent to give the product as a yellow oil (1.0 g, 47% yield). ^{1}H NMR (400 MHz, $CDCl_3$) δ 10.12 (s, 1H), 8.38 (s, 1H), 8.12 (d, J = 9.0 Hz, 1H), 7.98 (d, J = 9.0 Hz, 1H), 7.89 (d, J = 8.2 Hz, 1H), 7.69 (d, J = 8.9 Hz, 1H), 7.52 (d, J = 9.0 Hz, 1H), 7.44 (d, J = 9.0 Hz, 1H), 7.34 (t, J = 7.5 Hz, 1H), 7.30–7.20 (m, 3H), 7.12 (d, J = 8.5 Hz, 1H), 4.09–3.90 (m, 4H), 1.50–1.35 (m, 4H), 1.08–0.94 (m, 4H), 0.72–0.61 (m, 6H). ^{13}C{^{1}H} NMR (101 MHz, $CDCl_3$) δ 192.16, 157.33, 154.47, 137.66, 134.91, 133.96, 132.04, 131.01, 129.46, 129.18, 127.98, 127.94, 126.55, 126.25, 125.03, 123.48, 123.02, 120.89, 119.45, 115.86, 115.40, 69.19, 68.95, 31.34, 31.17, 18.74, 18.69, 13.54, 13.52. HRMS-ESI, calcd. for $C_{29}H_{31}O_3$ $(M+H)^+$: 427.2268; found 427.2282.

Synthesis of (1*R*)-2,2′-bis(butyloxy)-[1,1′-binaphthalene]-6-methanol (2d)

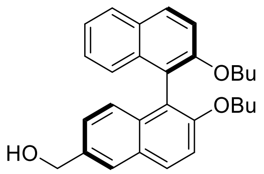

In a 50 mL round bottom flask, at room temperature, 2c (1 g, 2.34 mmol) was dissolved in a 1:1 mixture of THF and methanol (30 mL). To this mixture sodium borohydride (177 mg, 4.68 mmol, 2 equiv) was added. This mixture was stirred for 1 h before adding 20 mL of water, then stirred for an additional 30 min. Excess solvent was removed via rotary evaporator and washed using ethyl acetate. The mixture was dried over Na_2SO_4 and purified using a flush column, with 10% ethyl acetate in hexanes as an eluent, to yield the desired product as a colorless oil (0.8 g, 80% yield). ^{1}H NMR (400 MHz, $CDCl_3$) δ 7.99–7.91 (m, 2H), 7.91–7.81 (m, 2H), 7.44 (dd, J = 9.0, 2.0 Hz, 2H), 7.33 (ddd, J = 8.1, 6.6, 1.3 Hz, 1H), 7.26–7.10 (m, 4H), 4.82 (s, 2H), 3.96–3.76 (m, 4H), 1.51–1.32 (m, 4H), 1.10–0.89 (m, 4H), 0.86–0.66 (m, 6H). ^{13}C{^{1}H} NMR (101 MHz, $CDCl_3$) δ 154.74, 154.55, 135.71, 134.21, 133.83, 129.23, 129.06, 128.98, 127.78, 126.07, 126.05, 125.69, 125.53, 125.42, 123.38, 120.72, 120.58, 116.09, 115.83, 69.41, 69.37, 65.63, 31.41, 31.39, 18.75, 18.73, 13.59, 13.56 (one sp^2 carbon signal was not observed, likely due to signal overlap). HRMS-ESI, calcd. for $C_{29}H_{32}O_3K$ $(M+K)^+$: 467.1983; found 467.1995.

Synthesis of diethyl *P*-(1*R*)-2,2′-bis(butyloxy)-[1,1′-binaphthalene]-6-yl-methylene-phosphonate (2e)

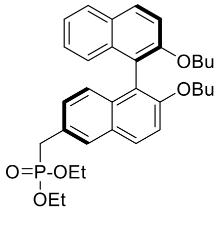

Triethyl phosphite (2 mL) and 2d (0.8 g, 1.87 mmol) were added to a 25 mL round bottom flask. The solution was cooled to 0 °C in an ice bath, followed by the addition of I_2 (0.48 g, 2.34 mmol, 1.0 equiv). Upon completion of the reaction as monitored by TLC, excess triethyl phosphite was removed by distillation under reduced pressure (100 °C, 1–2 Torr). The crude product was purified by flash column chromatography using 3:2 ethyl acetate/hexanes as the eluent to afford the desired product as a yellow oil (0.4 g, 0.73 mmol, 39% yield). ^{1}H NMR (400 MHz, $CDCl_3$) δ 7.98–7.83 (m, 3H), 7.82–7.77 (m, 1H), 7.42 (dd, J = 9.0, 3.2 Hz, 2H), 7.32 (dt, J = 7.6, 1.2 Hz, 1H), 7.21 (dt, J = 7.6, 1.2 Hz, 1H), 7.17–7.08 (m, 3H), 4.20–3.87 (m, 8H), 3.28 (d, J = 20 Hz, 2H), 1.49–1.32 (m, 4H), 1.32–1.19 (m, 6H), 1.08–0.94 (m, 4H), 0.66 (t, J = 7.4 Hz, 3H), 0.65 (t, J = 7.4 Hz, 3H). ^{13}C{^{1}H} NMR (101 MHz, $CDCl_3$) δ 154.52, 134.19, 133.13 (d, $J_{C\text{-}P}$ = 1 Hz), 129.25, 129.22, 129.20, 129.04, 128.73, 128.41 (d, $J_{C\text{-}P}$ = 8 Hz), 128.08 (d, $J_{C\text{-}P}$ = 5 Hz), 127.76, 126.29 (d, $J_{C\text{-}P}$ = 10 Hz), 125.99, 125.85 (d, $J_{C\text{-}P}$ = 3 Hz), 125.47, 123.35, 120.56, 120.51, 116.12, 115.77, 69.40, 69.37, 62.12 (d, $J_{C\text{-}P}$ = 6 Hz), 62.07 (d, $J_{C\text{-}P}$ = 6 Hz), 33.65 (d, $J_{C\text{-}P}$ = 137 Hz), 31.39, 31.38, 18.74, 18.72, 16.39 (d, $J_{C\text{-}P}$ = 6 Hz), 13.58, 13.56 (Note that the ethyl carbons are in principle diastereotopic, which results in the inequivalence of the two ethyl CH_2 ^{13}C resonances). ^{31}P NMR (162 MHz, $CDCl_3$) δ 26.73. HRMS-ESI, calcd. for C33H41O5P (M+H)$^+$: 549.2764; found 549.2788.

Synthesis of *P*-(1*R*)-2,2′-bis(butyloxy)-[1,1′-binaphthalene]-6-yl-methylene-phosphonic acid (BPA2)

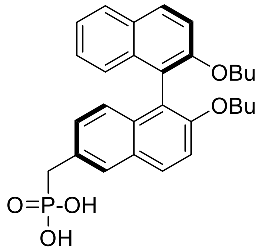

To a 25 mL round bottom flask, 2e (0.4 g, 0.73 mmol) and DCM (10 mL) was added. The mixture was cooled in an ice bath before the addition of TMSBr (0.6 mL, 0.71g, 4.62 mmol, 6 equiv). The mixture was left to stir and come to room temperature overnight. Excess solvent was removed using a rotary evaporator. Methanol (0.6 mL) and water (3.5 mL) were added to the mixture and the new solution was stirred for 30 min. Excess solvent was again removed using a rotary evaporator before adding water (6 mL). The solution was filtered and dried under vacuum to produce the product as a brown solid product (300 mg, 0.61 mmol, 84%). The product is soluble in DCM, toluene, diethyl ether, and acetonitrile, but insoluble in hexanes. ^{1}H NMR (400 MHz, DMSO-d_6) δ 8.02 (d, J = 9.0 Hz, 1H), 7.93 (t, J = 8.4 Hz, 2H), 7.75 (d, J = 3.0 Hz, 1H), 7.55 (dd, J = 14.3, 9.0 Hz, 2H), 7.31 (t, J = 7.5 Hz, 1H), 7.21 (t, J = 7.6 Hz, 1H), 7.12 (d, J = 8.8 Hz, 1H), 6.91 (d, J = 8.5 Hz, 1H), 6.82 (d, J = 8.7 Hz, 1H), 5.17–4.88 (br, 2H), 3.96 (t, J = 6.5 Hz, 2H), 3.94 (t, J = 6.5 Hz, 2H), 3.05 (d, J = 21.2 Hz, 2H), 1.43–1.27 (m, 4H), 1.14–0.89 (m, 4H), 0.66 (t, J = 7.4 Hz, 3H), 0.31 (t, J = 7.2 Hz, 3H). ^{13}C{^{1}H} NMR (101 MHz, DMSO-d_6) δ 154.52, 154.13, 134.01, 132.53 (d, J = 2 Hz), 129.57, 129.54, 129.48, 129.22, 129.14, 129.04, 128.49, 128.41, 128.37, 126.51, 125.09, 124.74, 123.67,

119.84, 119.69, 115.96 (d, J = 3 Hz), 68.70, 35.73 (d, J = 131 Hz), 31.36, 31.35, 18.74, 18.70, 13.93, 13.88 (1 sp^3 signal not observed, presumably due to overlap). ^{31}P NMR (162 MHz, DMSO-d_6) δ 21.30. HRMS-ESI, calcd. for $C_{29}H_{33}O_5P$ $(M\text{-}H)^-$: 491.1993; found 490.1997.

Synthesis of diethyl *P*-[[(1*R*)-2′-methoxy[1,1′-binaphthalene]-2-yl]oxy-3,1-propyl]phosphonate (3b)

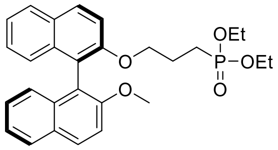

To a 100 mL flask, dry DMSO (40 mL) was added to dissolve (*R*)-2-hydroxy-2′-methoxy-1,1′-binaphthyl (3a, 2.0 g, 6.7 mmol). Cesium carbonate (6.6 g, 20 mmol) and diethyl *P*-(3-bromopropyl)phosphonate (3.3 g, 13.4 mmol) were added. The reaction mixture was stirred for 12 h at 90 °C under nitrogen, and the reaction conversion was followed by TLC. Once the reaction was determined to be complete by TLC, the mixture was cooled to room temperature. The crude reaction mixture was poured into an equal volume of brine and extracted with DCM. The combined organic portions were dried with Na_2SO_4, filtered, and concentrated. The crude was purified by silica gel chromatography (10% ethyl acetate in DCM) to elute product 3b as a colorless sticky oil (3.0 g, 94%). ^1H NMR (400 MHz, $CDCl_3$) δ 8.02–7.93 (m, 2H), 7.88 (dddt, J = 8.0, 7.4, 1.2, 0.6 Hz, 2H), 7.45 (dd, J = 16.6, 9.0 Hz, 2H), 7.34 (dddd, J = 9.4, 8.1, 6.7, 1.3 Hz, 2H), 7.24 (dddd, J = 8.9, 7.8, 6.6, 1.3 Hz, 2H), 7.18 (ddd, J = 8.5, 1.4, 0.7 Hz, 1H), 7.13 (ddt, J = 8.5, 1.5, 0.8 Hz, 1H), 4.22–3.93 (m, 2H), 3.93–3.78 (m, 4H), 3.79 (s, 3H), 1.84–1.64 (m, 2H), 1.40–1.24 (m, 2H), 1.25-1.18 (m, 6H). $^{13}C\{^1H\}$ NMR (101 MHz, $CDCl_3$) δ 154.87, 153.98, 134.05, 129.48, 129.41, 129.33, 129.11, 127.96, 127.85, 126.34, 125.35, 125.24, 123.75, 123.50, 120.52, 119.43, 115.70, 113.85, 69.16 (d, $J_{C\text{-}P}$ = 18 Hz), 61.28 (d, $J_{C\text{-}P}$ = 6 Hz), 61.25 (d, $J_{C\text{-}P}$ = 6 Hz), 56.67, 22.76 (d, $J_{C\text{-}P}$ = 5 Hz), 21.27 (d, $J_{C\text{-}P}$ = 140 Hz), 16.34 (d, $J_{C\text{-}P}$ = 6 Hz) (Note that the ethyl carbons are in principle diastereotopic, which results in the inequivalence of the two ethyl CH_2 ^{13}C resonances; two fewer aromatic resonances than expected are observed, presumably due to overlap). ^{31}P NMR (162 MHz, $CDCl_3$) δ 31.98. HRMS-ESI, calcd. for $C_{28}H_{31}O_5P$ $(M+H)^+$: 479.1982; found 479.2013.

Synthesis of *P*-[[(1*R*)-2′-methoxy[1,1′-binaphthalene]-2-yl]oxy-3,1-propyl]phosphonic acid (BPA3)

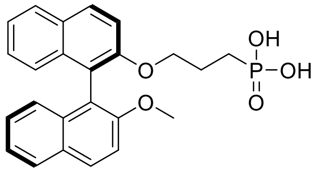

To the dichloromethane (50 mL) solution of 3b (2.1 g, 4.48 mmol) at 0 °C was added trimethylsilyl bromide (3.0 mL, 22.2 mmol). The result mixture was stirred overnight and slowly warmed to room temperature. After TLC indicates the hydrolysis is done, MeOH (20 mL) and water (100 mL) were added and stirred for 30 min. Volatile solvents were removed on rotavapor. The resulting water solid was washed with water and ether to get the desired product (1.7 g, 90%) as a white solid. ^1H NMR (400 MHz, 0.4 mL DMSO-d_6 and 5

μL D_2O) *δ* 8.10–8.01 (m, 2H), 7.94 (d, *J* = 8.1 Hz, 2H), 7.59 (t, *J* = 8.9 Hz, 2H), 7.32 (q, *J* = 6.7 Hz, 2H), 7.22 (t, *J* = 7.7 Hz, 2H), 6.89 (d, *J* = 8.5 Hz, 2H), 4.04 (t, *J* = 6.5 Hz, 2H), 3.73 (s, 3H), 1.60 (m, 2H), 1.27 (dt, *J* = 15.9, 7.6 Hz, 2H). $^{13}C\{^1H\}$ NMR (101 MHz, 0.4 mL DMSO-d_6 and 5 μL D_2O) δ 154.91, 154.23, 133.92, 133.80, 129.82, 129.69, 129.25, 129.03, 128.48, 128.46, 126.77, 126.75, 124.94, 124.90, 123.86, 123.71, 119.60, 118.65, 116.01, 114.24, 69.15 (d, *J* = 18 Hz), 56.39, 24.07 (d, *J* = 144 Hz), 23.36 (d, *J* = 10 Hz). ^{31}P NMR (162 MHz, 0.4 mL DMSO-d_6 and 5 μL D_2O) *δ* 26.26. HRMS-ESI, calcd. for $C_{24}H_{23}O_5P$ $(M-H)^-$: 421.1210; found 421.1213. Note: D_2O is added to simplify the spectra, presumably by disrupting hydrogen-bonded species assumed to be responsible for a signal at ca. 20 ppm in the ^{31}P NMR spectra.

NMR Spectra

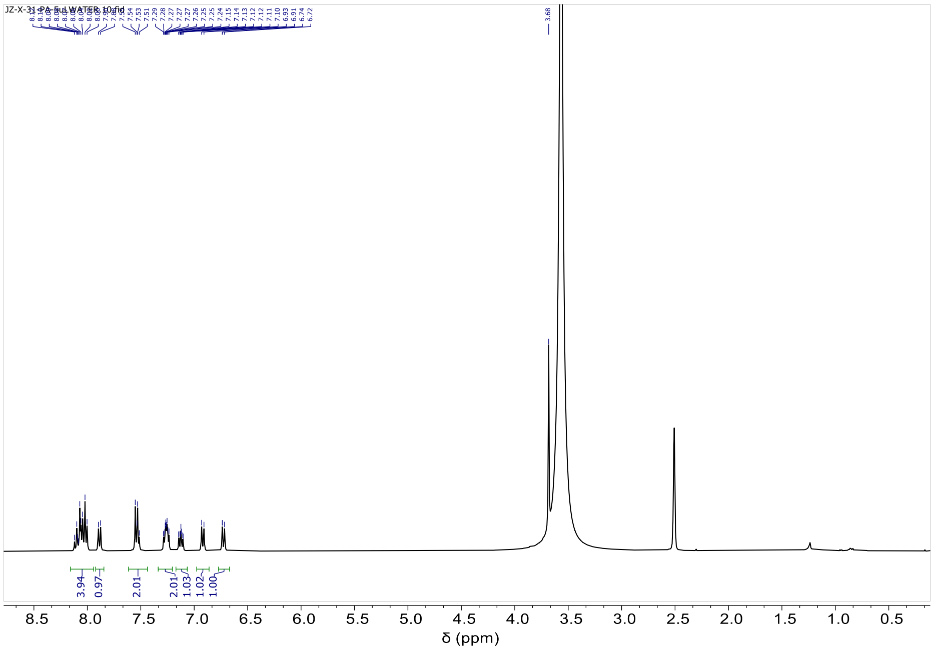

Figure S4: ^1H NMR spectrum of BPA1 in anhydrous DMSO-*d*6 (0.4 mL) and D_2O (5 μL).

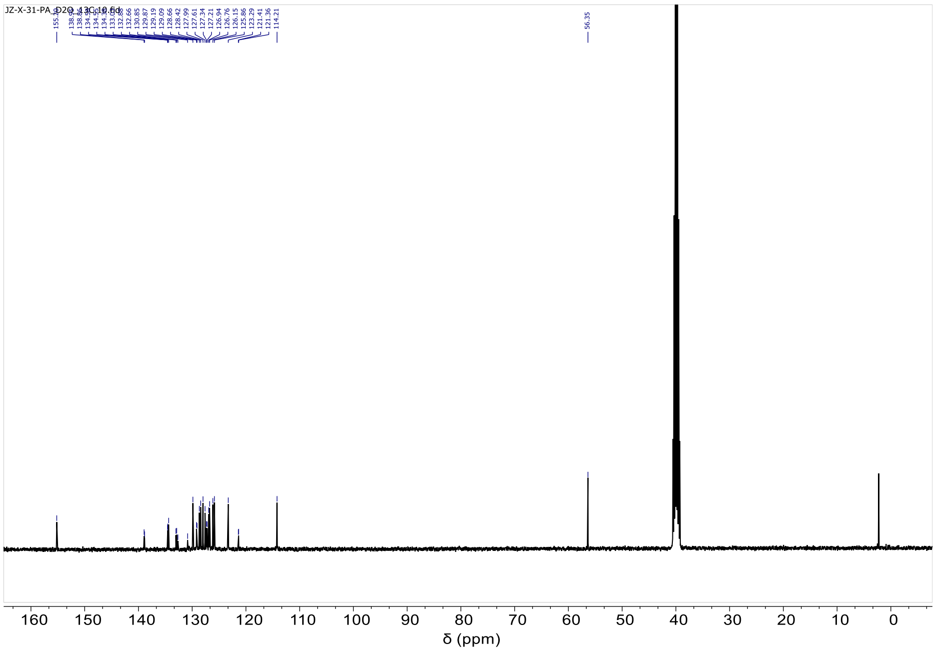

Figure S5: $^{13}C\{^{1}H\}$ NMR spectrum of BPA1 in anhydrous DMSO-d_6 (0.4 mL) and D_2O (5 µL).

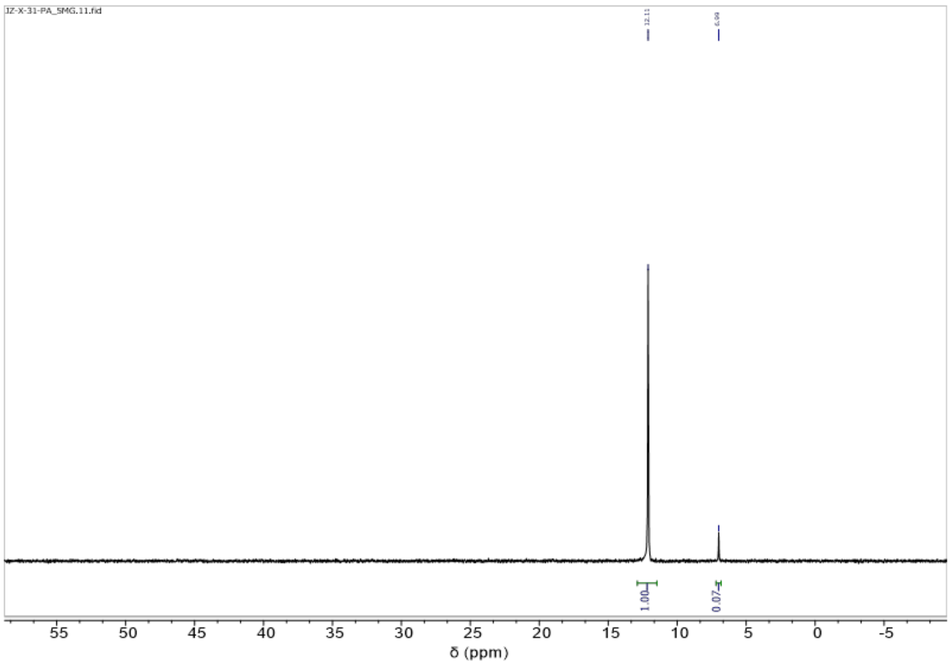

Figure S6: ^{31}P NMR spectrum of BPA1 in anhydrous DMSO-d_6.

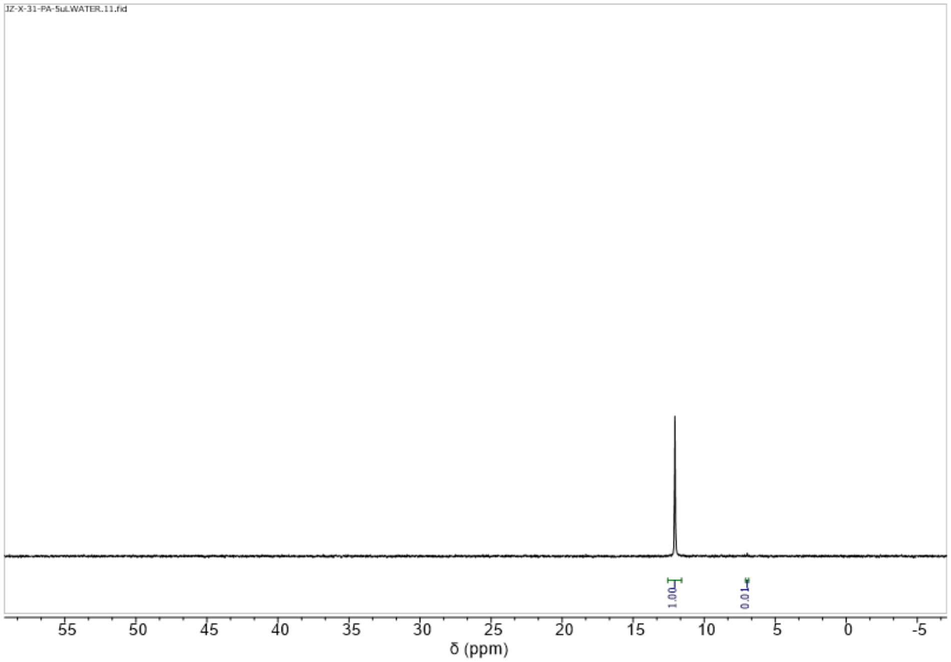

Figure S7: ^{31}P NMR spectrum of BPA1 in anhydrous DMSO-d_6 (0.4 mL) and D_2O (5 µL). The relative intensity of the signal at 6.99 ppm in this solvent mixture is significantly lower than what is seen in DMSO-d_6 along (Figure S6).

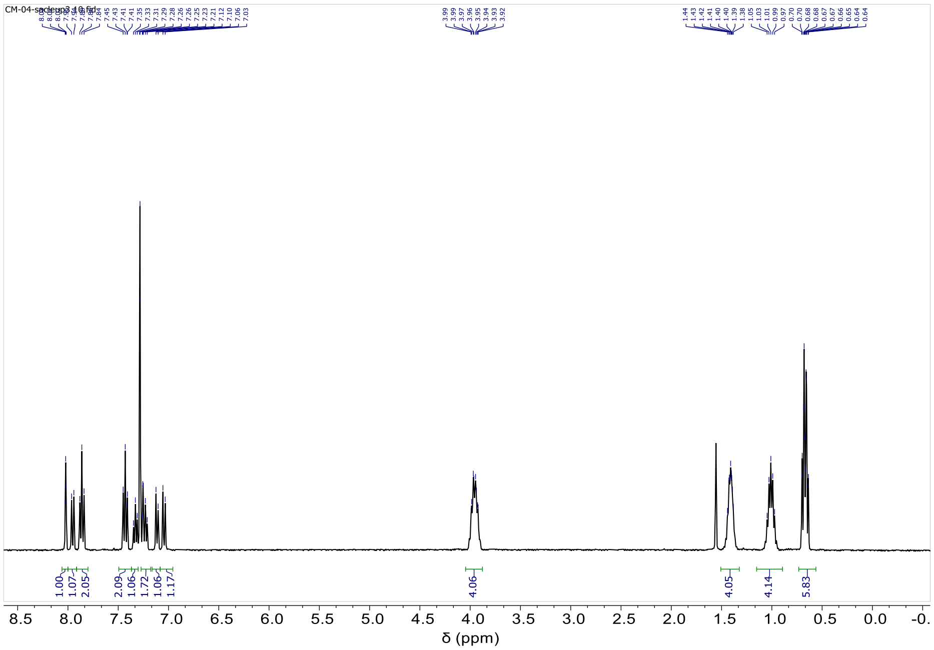

Figure S8: ^{1}H NMR spectrum of 2b in $CDCl_3$.

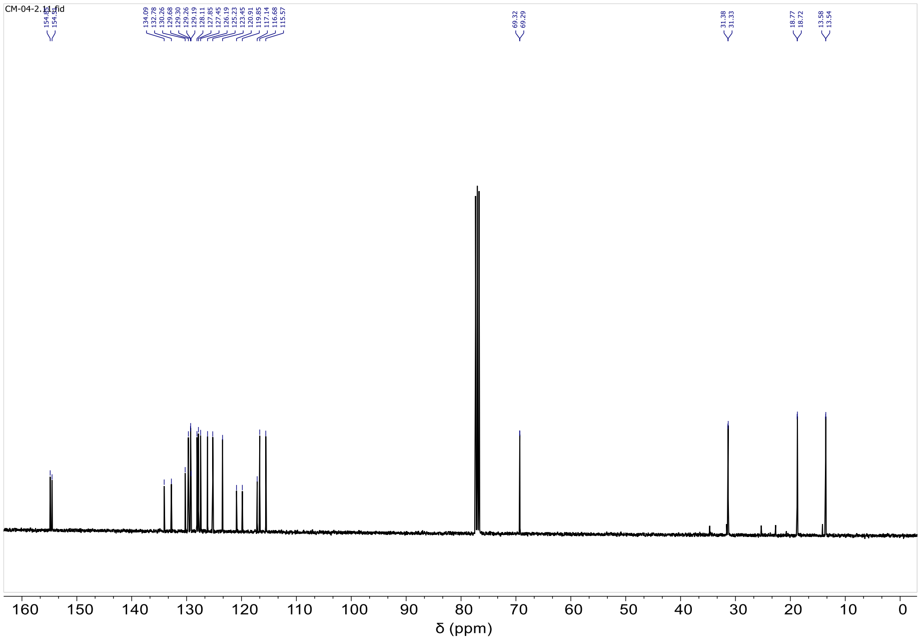

Figure S9: $^{13}C\{^{1}H\}$ NMR spectrum of 2b in $CDCl_3$.

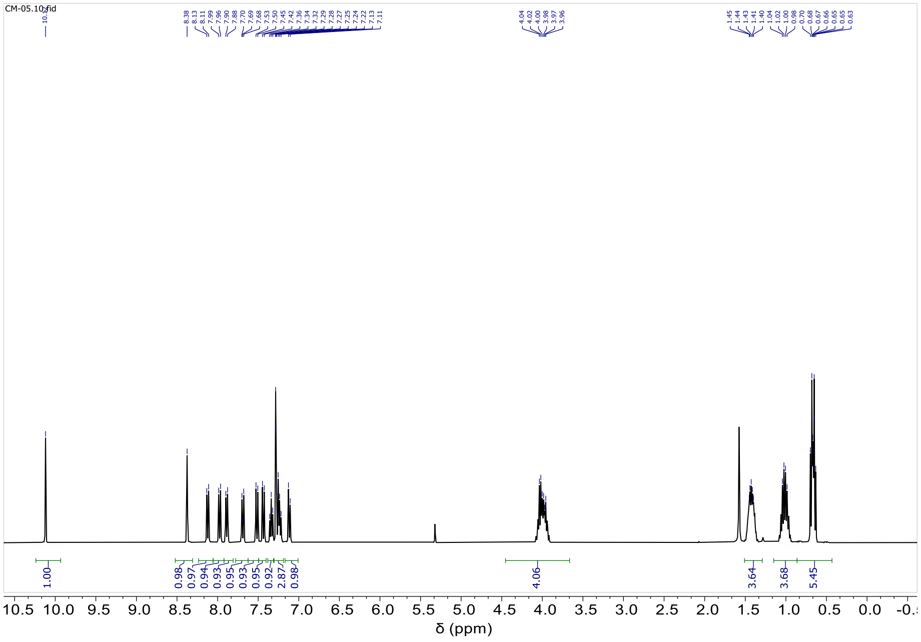

Figure S10: ^{1}H NMR spectrum of 2c in $CDCl_3$.

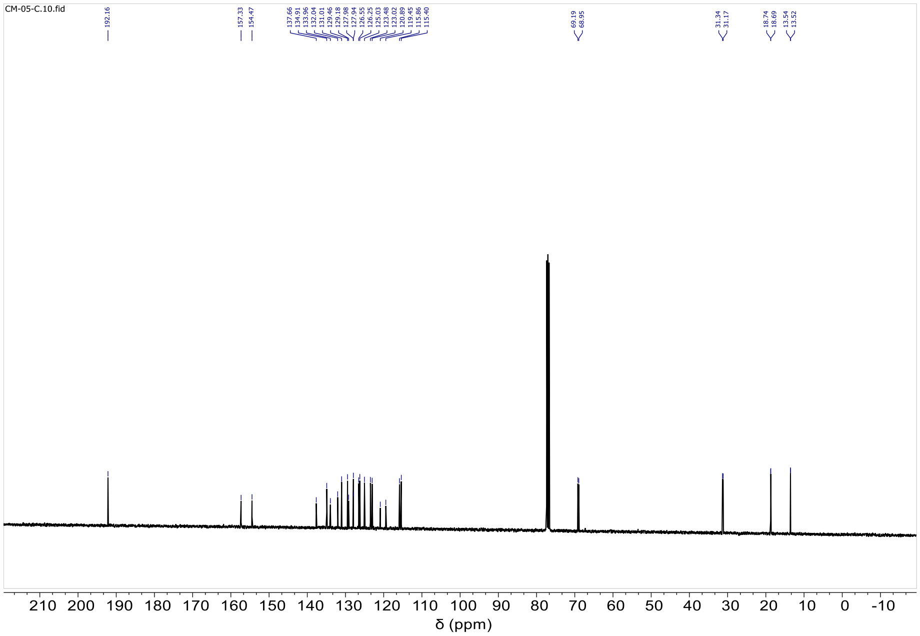

Figure S11: $^{13}C\{^1H\}$ NMR spectrum of 2c.

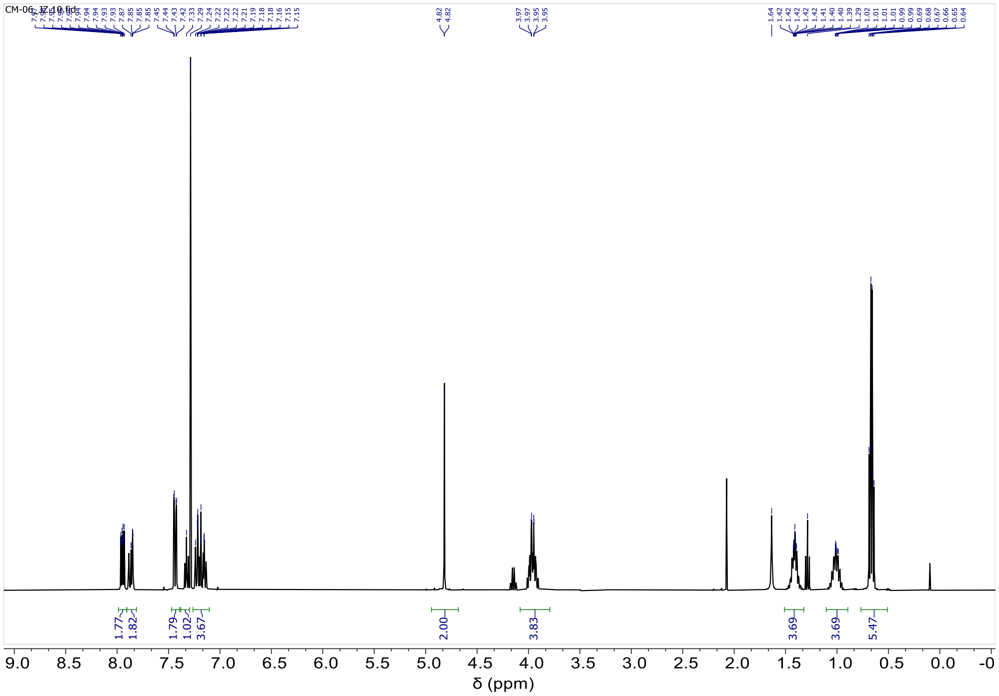

Figure S12: ^1H NMR spectrum of 2d in $CDCl_3$.

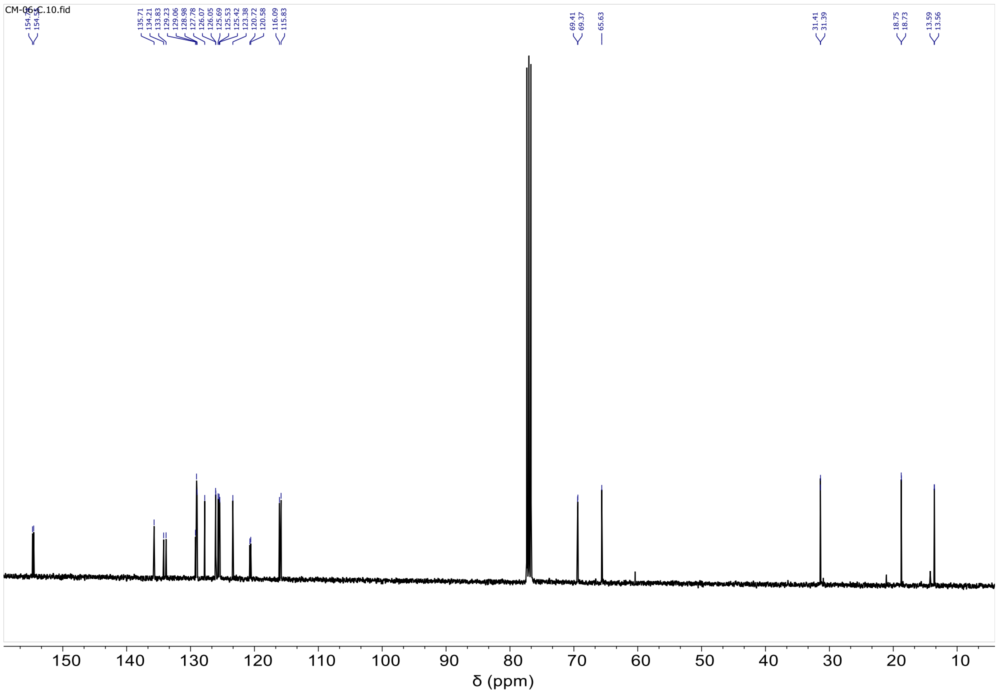

Figure S13: $^{13}C\{^1H\}$ NMR spectrum of 2d in $CDCl_3$.

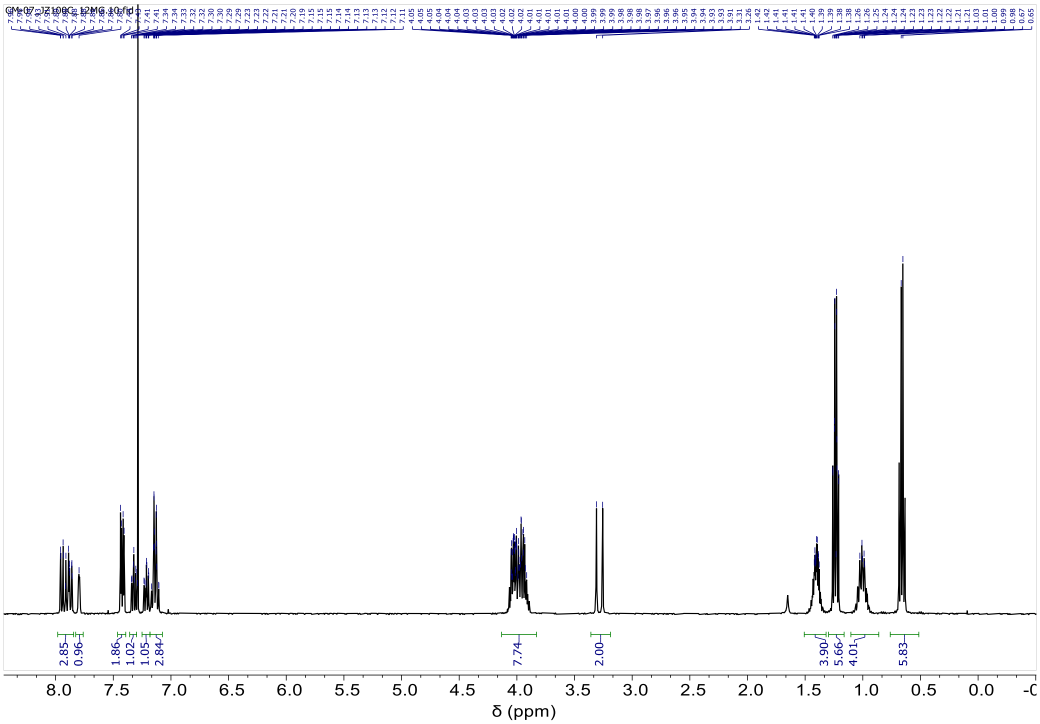

Figure S14: ^1H NMR spectrum of 2e in $CDCl_3$.

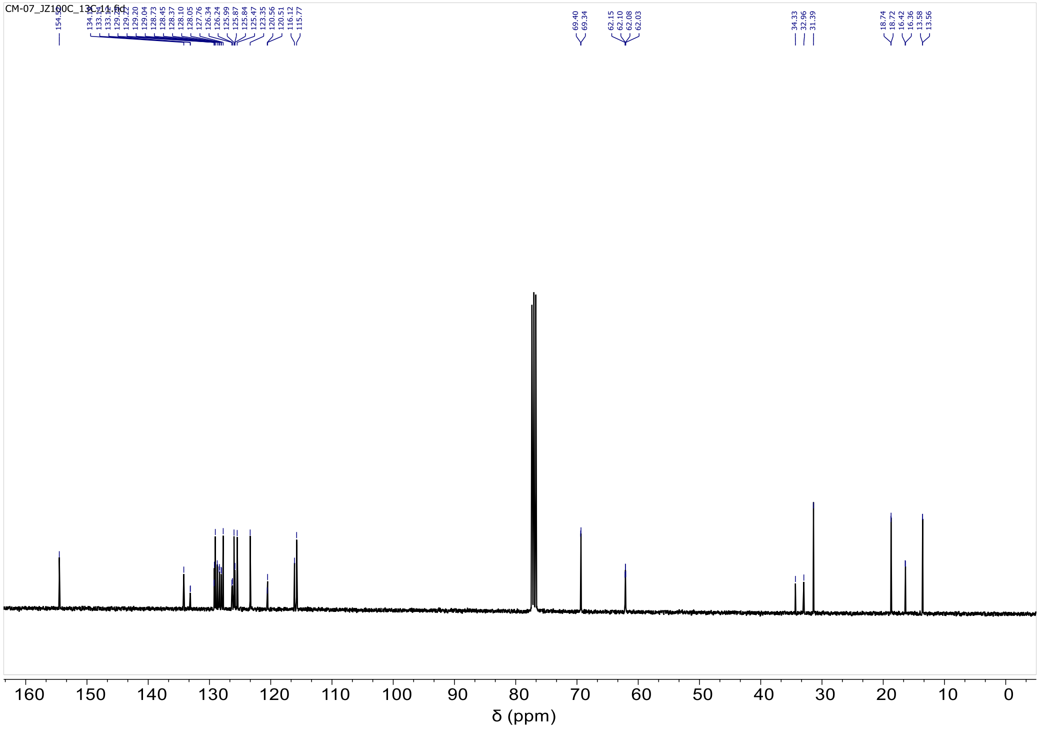

Figure S15: $^{13}C\{^{1}H\}$ NMR spectrum of 2e in $CDCl_3$.

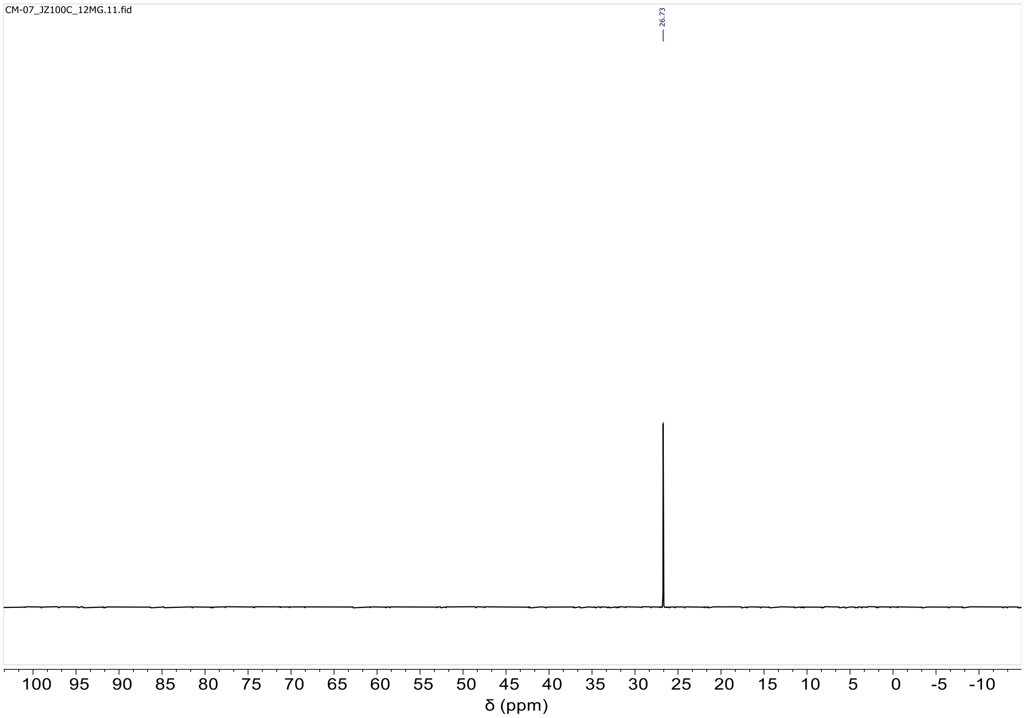

Figure S16: ^{31}P NMR spectrum of 2e in $CDCl_3$.

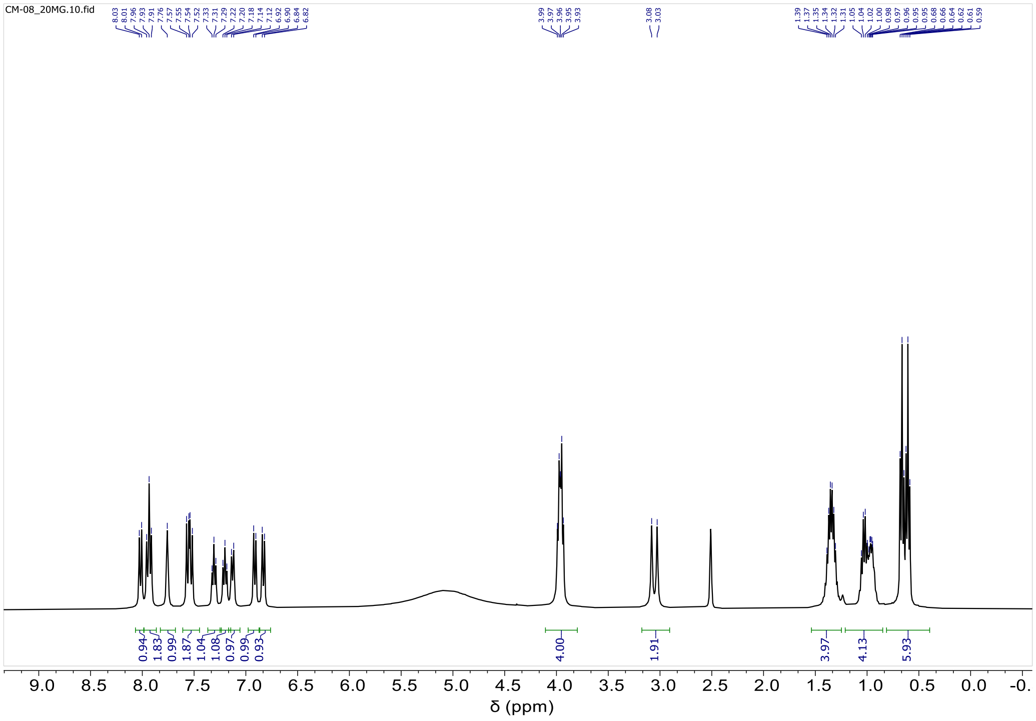

Figure S17: ^{1}H NMR spectrum of BPA2 in DMSO-d_6.

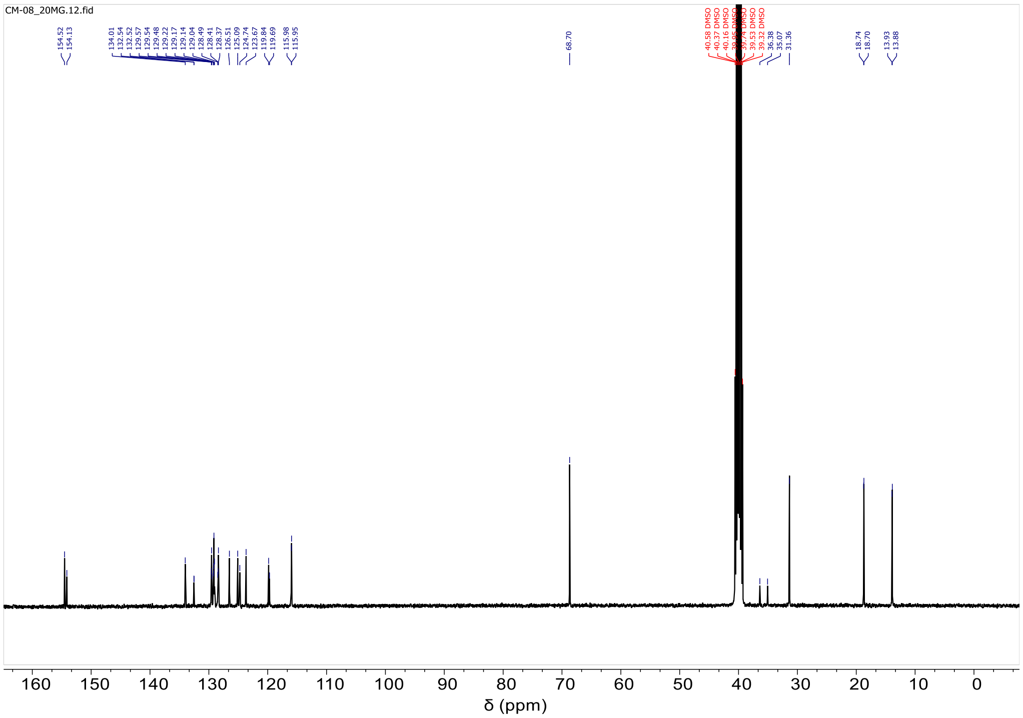

Figure S18: $^{13}C\{^{1}H\}$ NMR spectrum of BPA2 in DMSO-d_6.

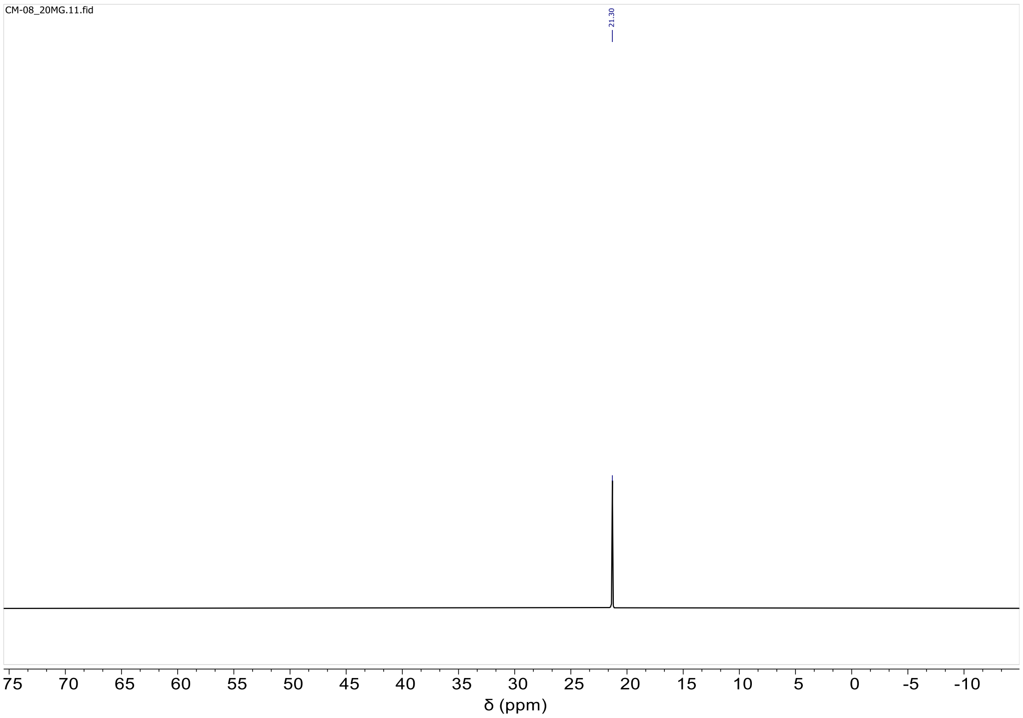

Figure S19: ^{31}P NMR spectrum of BPA2 in DMSO-d_6.

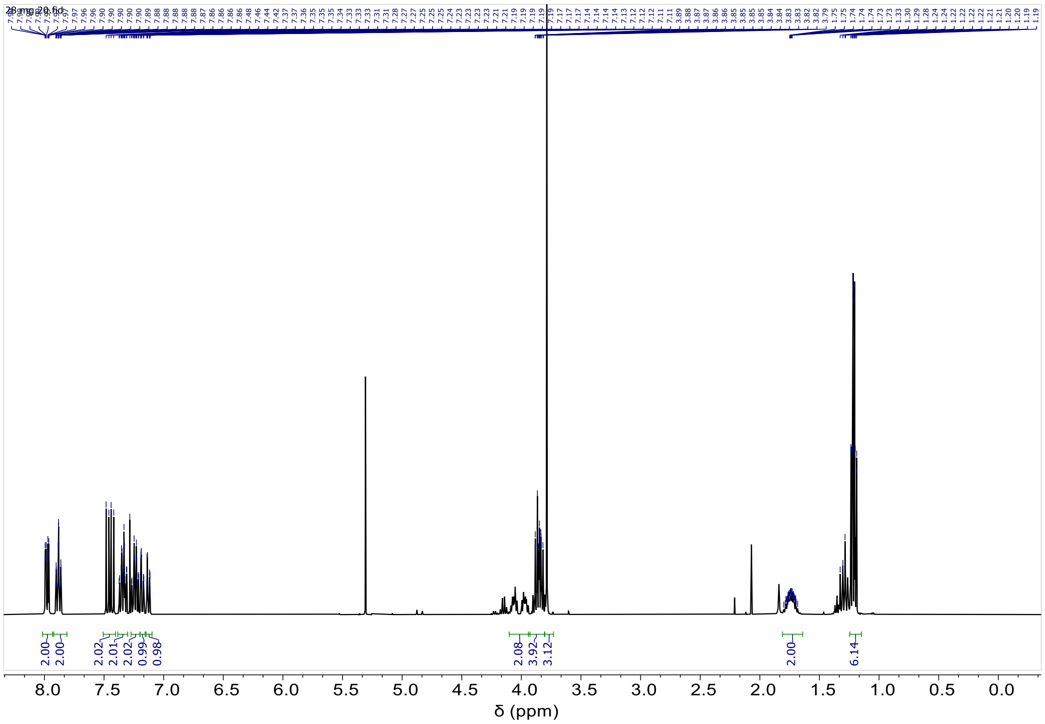

Figure S20: ^{1}H NMR spectrum of 3b in $CDCl_3$.

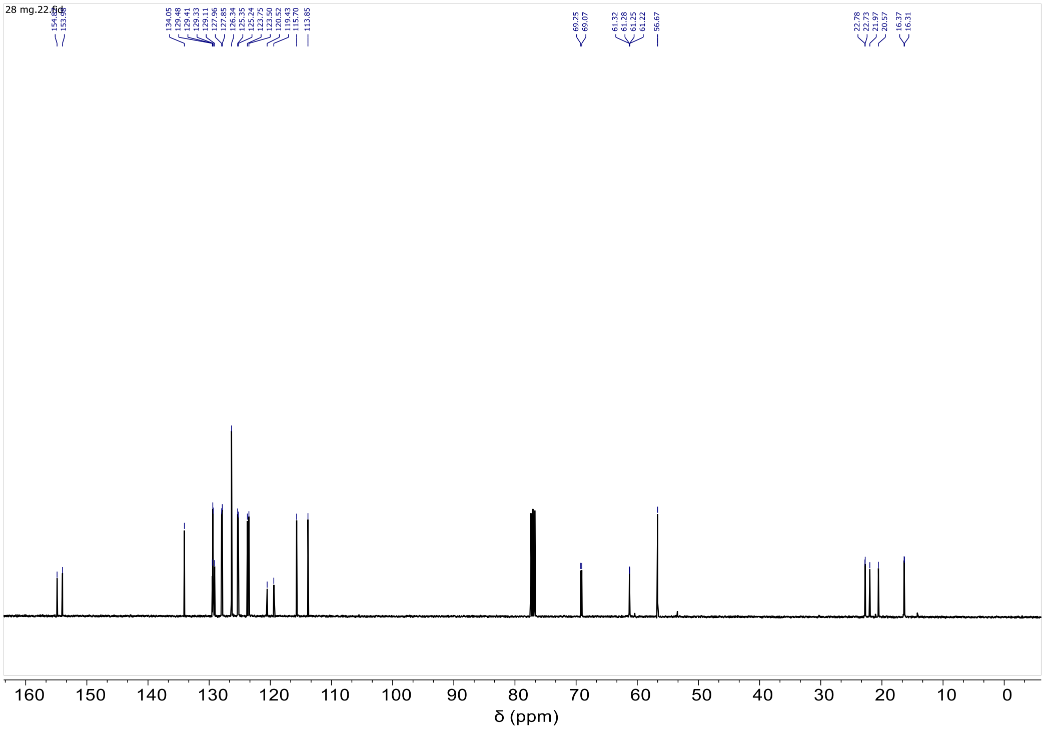

Figure S21: $^{13}C\{^{1}H\}$ NMR spectrum of 3b in $CDCl_3$.

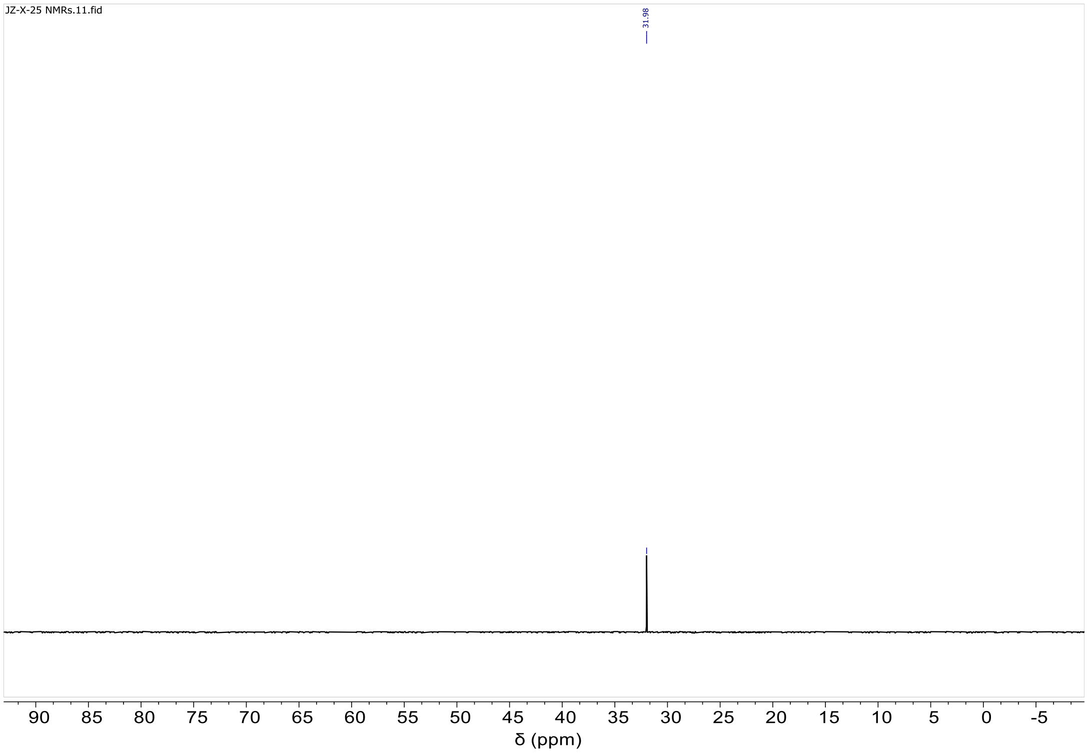

Figure S22: ^{31}P NMR spectrum of 3b in $CDCl_3$.

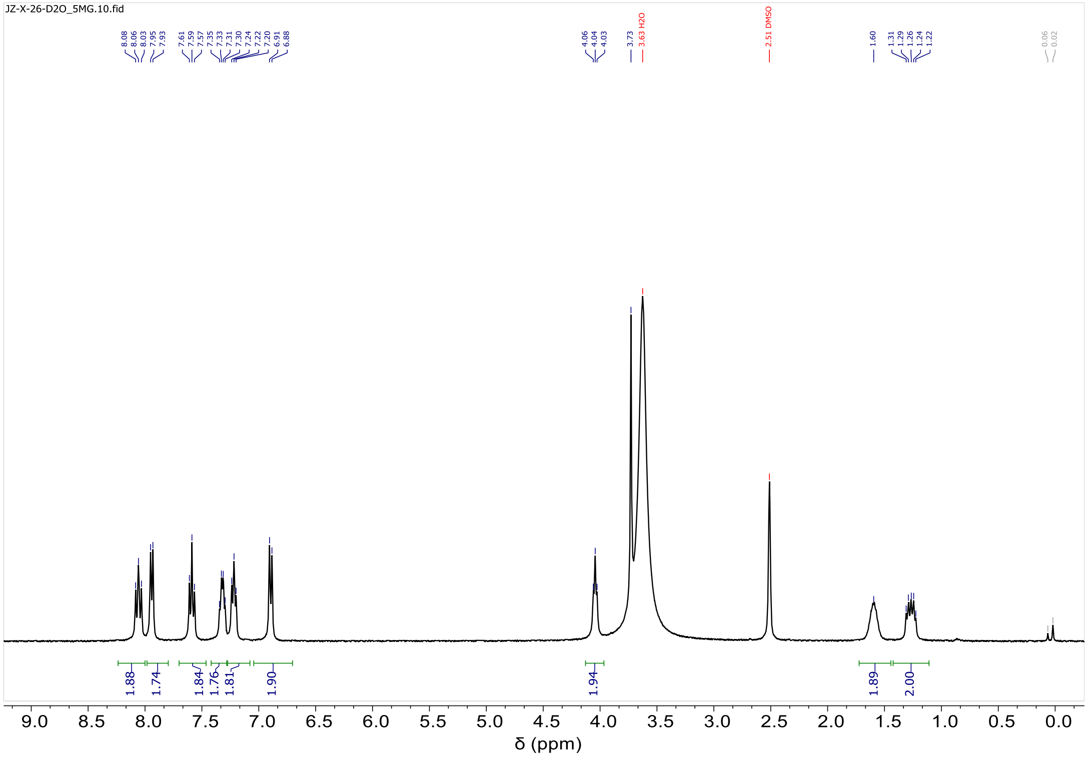

Figure S23: ^{1}H NMR spectrum of BPA3 in anhydrous DMSO-d_6 (0.4 mL) and D_2O (5 µL).

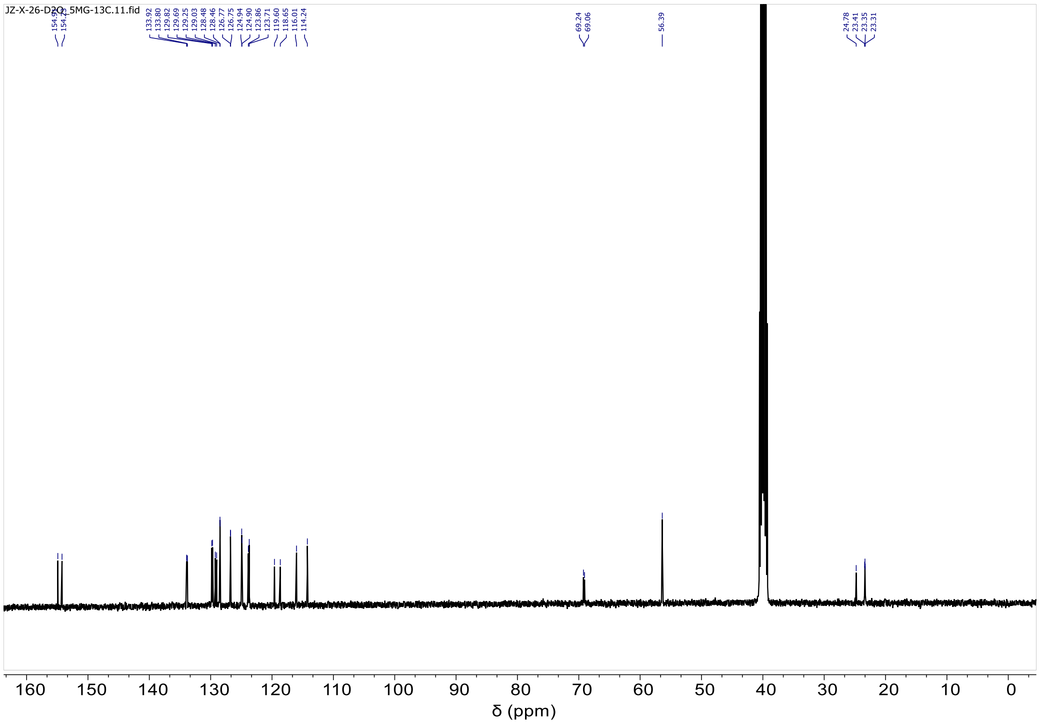

Figure S24: $^{13}C\{^{1}H\}$ NMR spectrum of BPA3 in anhydrous DMSO-d_6 (0.4 mL) and D_2O (5 µL).

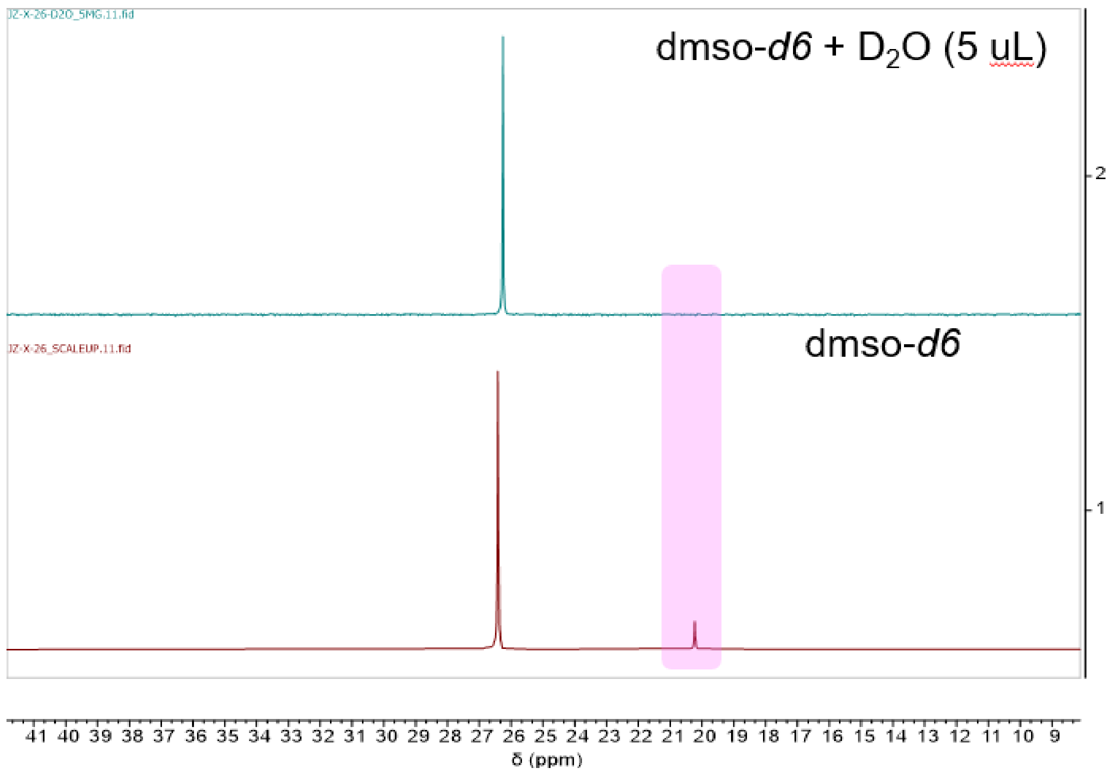

Figure S25: ^{31}P NMR spectrum of BPA3 in anhydrous DMSO-d_6 (bottom), and the mixture of anhydrous DMSO-d_6 (0.4 mL) and D_2O (5 μL) showing the much weaker signal at 20.26 ppm in the mixed solvent system.

Supporting data part 1: CD, XRD

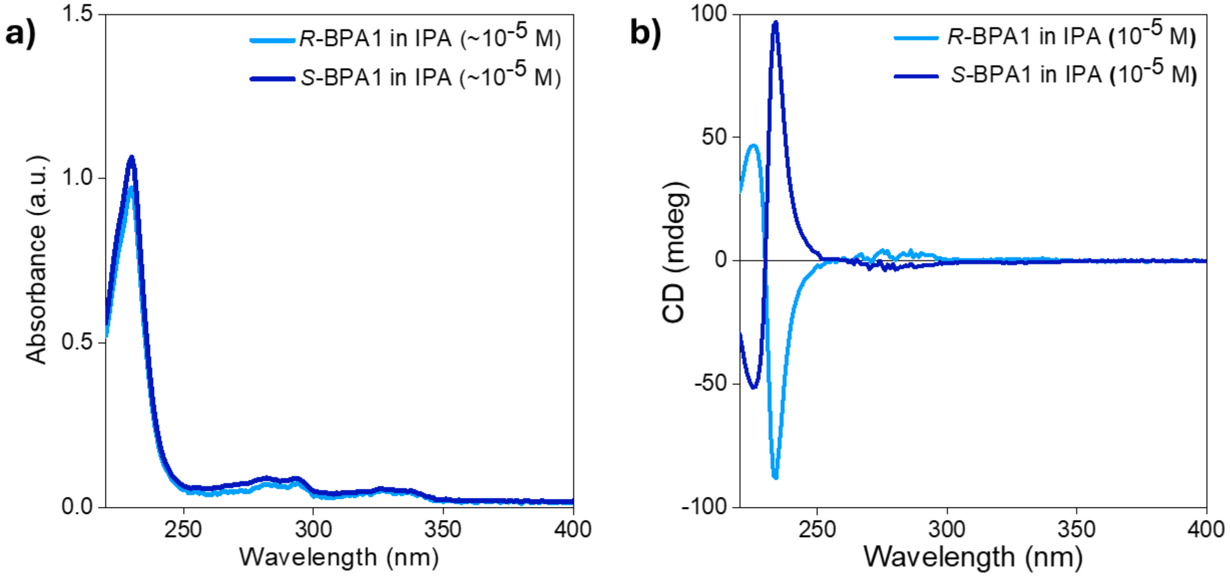

Figure S26: a) UV-Vis and **b)** CD spectra of *R*-/*S*-BPA1 in solution.

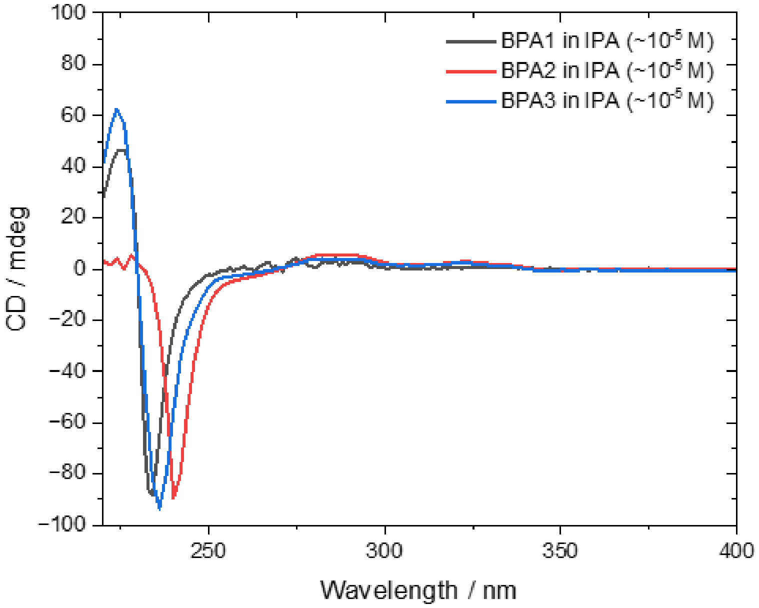

Figure S27: CD spectra of BPA1, BPA2 and BPA3.

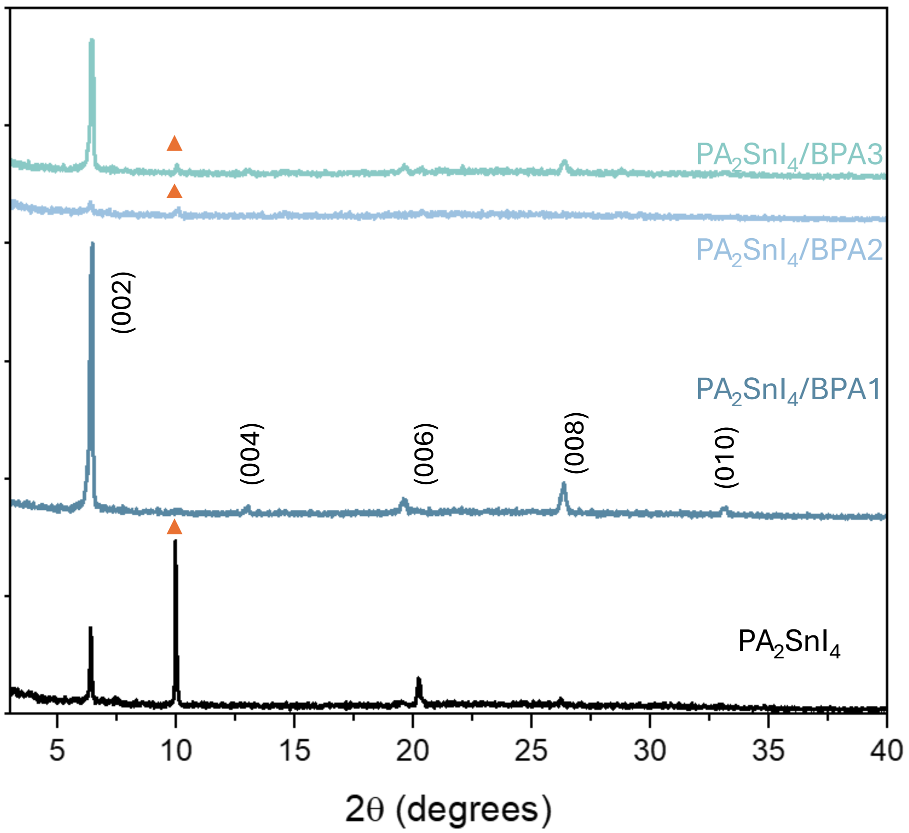

Figure S28: XRD pattern of PA_2SnI_4 film (black) after addition of 0.1 molecular fraction of BPA1(dark blue), -2 (blue) and -3 (green). The extra peak at 10 degrees in the control, BPA2 and BPA3 films are indicated with a red triangle.

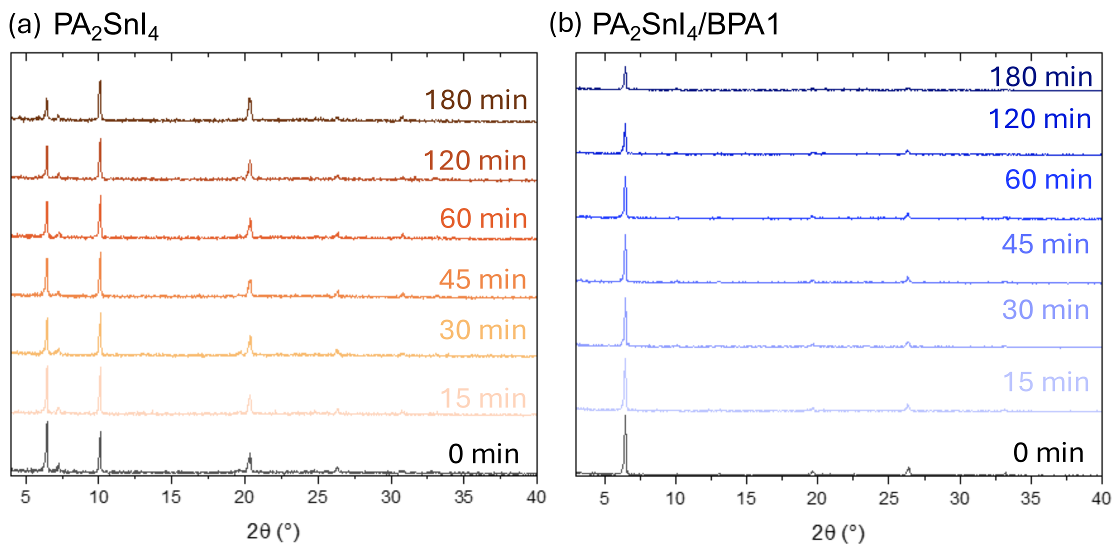

Figure S29: XRD pattern of **a)** the control PA_2SnI_4 film and **b)** with the addition of 0.1 molar equivalent of BPA1 measured overtime.

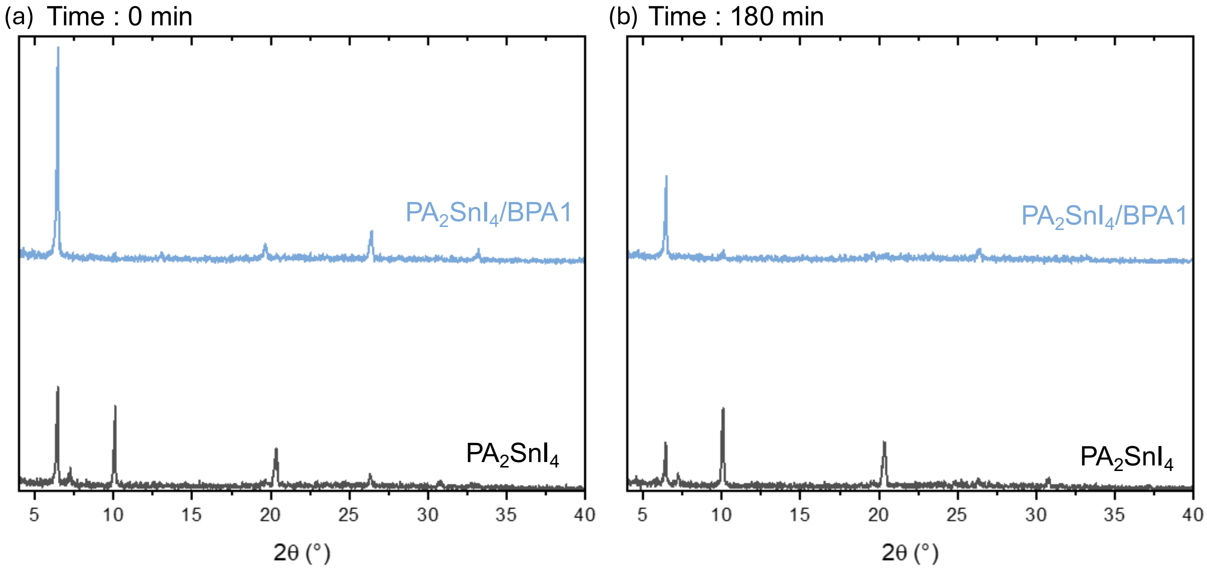

Figure S30: XRD pattern of the control PA_2SnI_4 and PA_2SnI_4/BPA1 at **a)** time 0 and **b)** after 180 min of aging in air.

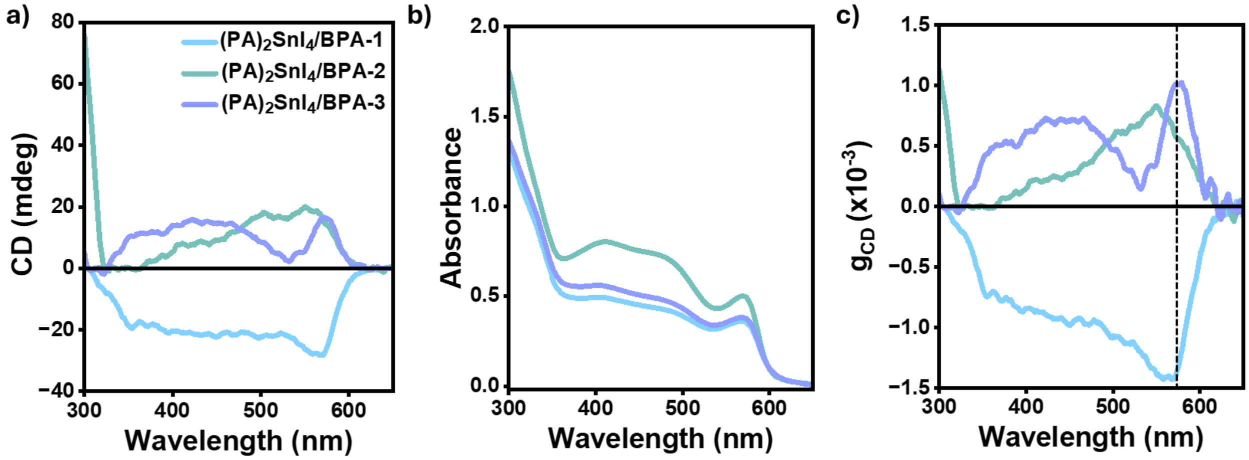

Figure S31: a) CD spectra of samples with addition of 0.1 molar equivalents of BPA1, 2 and 3. **b)** UV-Vis spectra and **c)** g_{CD}. The values highlighted at 570 nm is the g_{CD} reported in the main text Figure 2e.

Supplementary Note 2: Impact of Binaphthyl Chirality on the Phosphonic Acid Moiety

We assessed the relative influence of the chiral BINOL group on the phosphonic / phosphonate moiety via the solution ^{13}C signals of ethyl groups on the diethyl phosphonate precursors to BPA1-3 (1, 2e, and 3b respectively). Due to the chirality of the binaphthyl, the two ethyl CH_2 C nuclei in each compound are diastereotopic and thus, in principle, chemically inequivalent, as are the two ethyl CH_3 nuclei. The extent to which this chemical inequivalence results in distinguishable chemical shifts is therefore a crude measure of the effectively chiral character of the environment of these moieties. In the case of compound 1, two CH_2 and two CH_3 resonances are seen due to the proximity to the BINOL, whereas in 2e and 3b two CH_2 resonances are seen but only one CH_3 resonance is observed. Furthermore, the chemical shift difference of stereogenic methylene carbons in compound 1 (0.14 ppm) is significantly larger than those in compound 2e (0.05 ppm) and 3b (0.03 ppm).

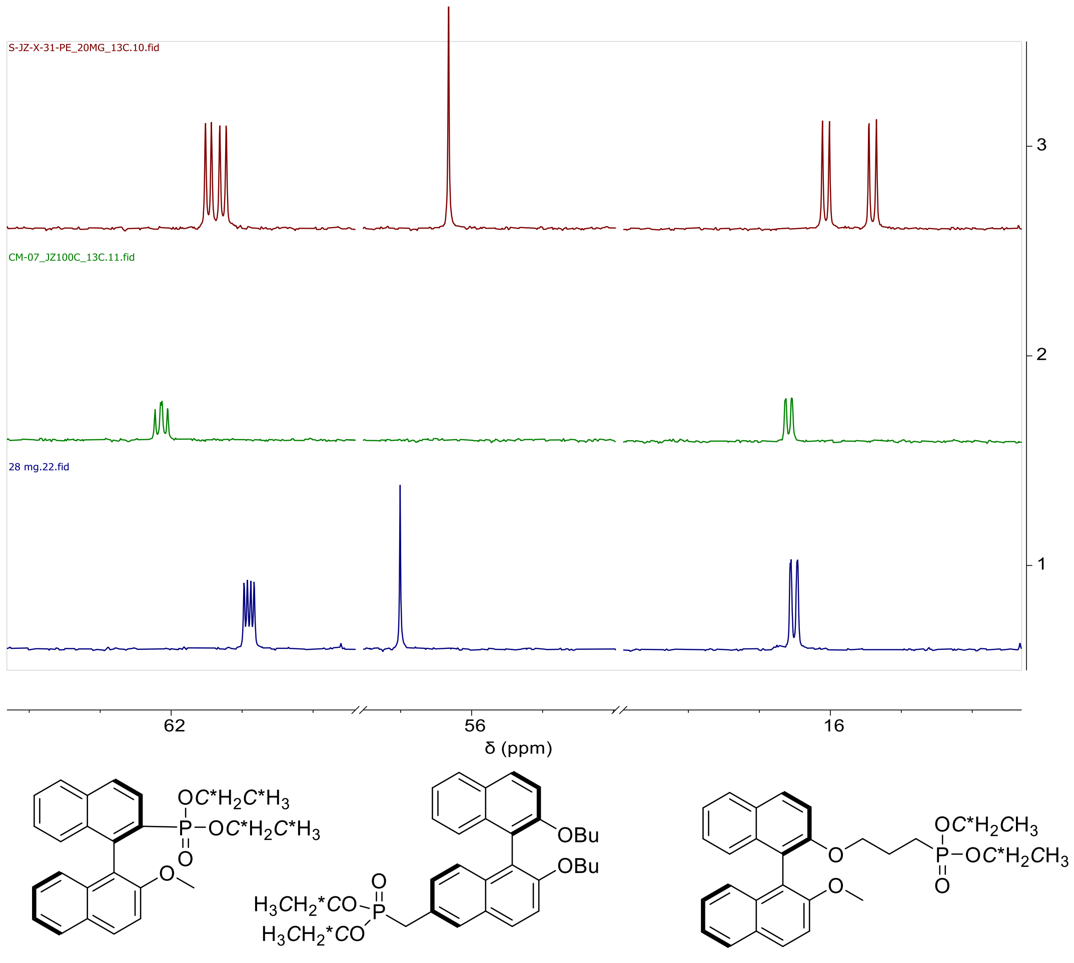

1 **2e** **3b**

Figure S32: Partial $^{13}C\{^1H\}$ NMR spectra of phosphonates showing the ethyl CH_2 and CH_3 resonances: compound 1 (top, 61.65 (d, $^2J_{C-P}$ = 6 Hz), 61.51 (d, $^2J_{C-P}$ = 6 Hz), 16.04 (d, $^3J_{C-P}$ = 6 Hz), 15.61 (d, $^3J_{C-P}$ = 6

Hz)); 2e (middle, 62.12 (d, $^2J_{C\text{-}P}$ = 6 Hz), 62.07 (d, $^2J_{C\text{-}P}$ = 6 Hz), 16.39 (d, $^3J_{C\text{-}P}$ = 6 Hz)); and 3b (bottom, 61.28 (d, $^2J_{C\text{-}P}$ = 6 Hz), 61.25 (d, $^2J_{C\text{-}P}$ = 6 Hz), 16.34 (d, $^3J_{C\text{-}P}$ = 6 Hz)).

Supporting data part 2: CD, XRD, SEM, ToF-SIMS, THz

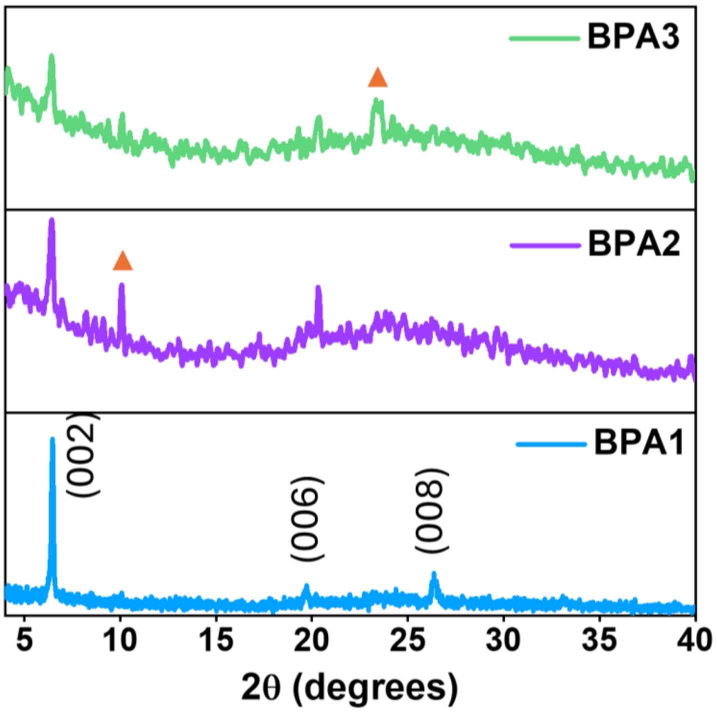

Figure S33: XRD pattern of PA_2SnI_4 films with addition of 0.3 molar equivalents of BPA1, 2 and 3. The secondary phases extra peak at 10 degrees in the BPA2 and 23 degrees for BPA3 films are indicated with a red triangle.

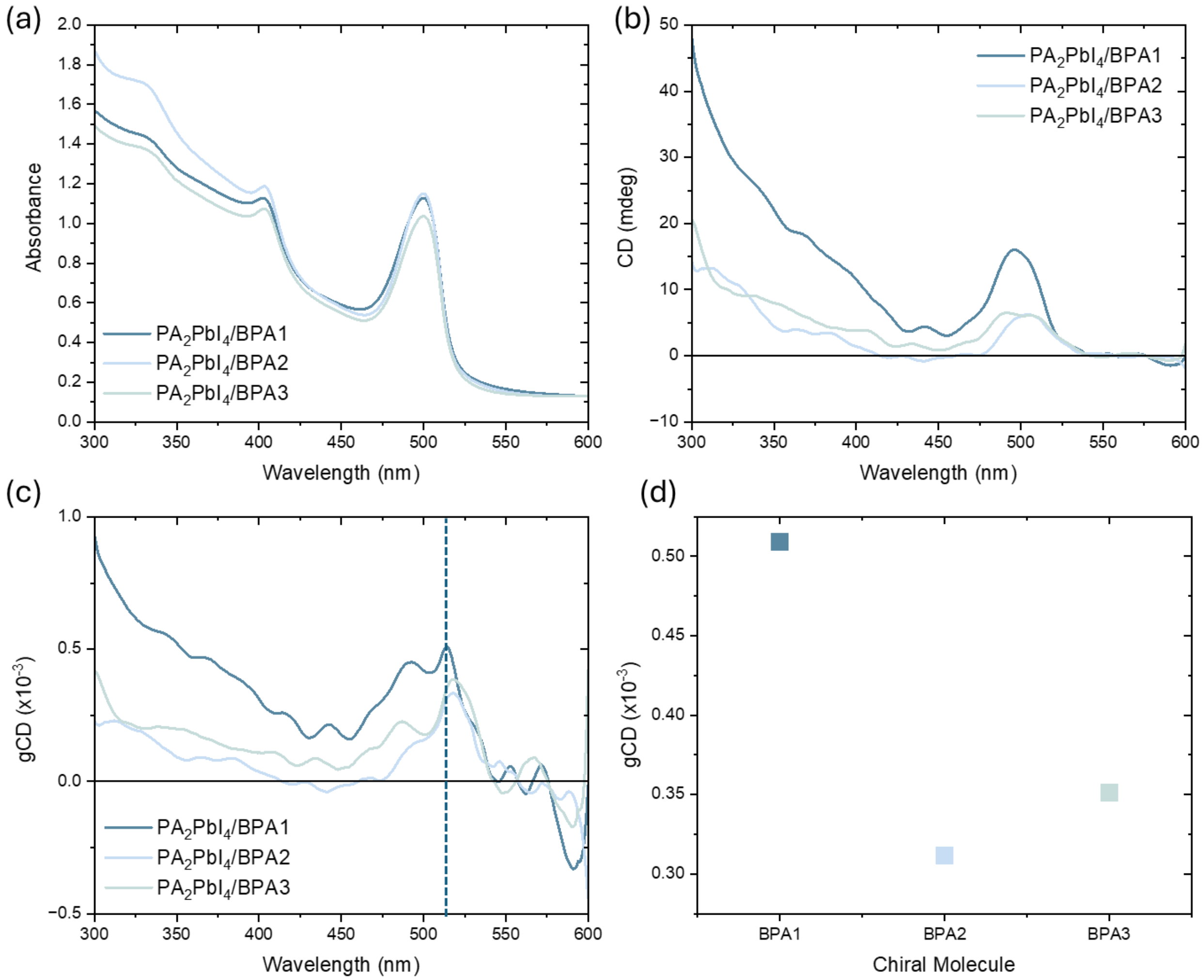

Figure S34: **a)** UV-Vis spectra of PA_2PbI_4 perovskite films with addition of 0.1 molar equivalents of BPA1 (dark blue), BPA2 (blue), BPA3 (green). **b)** CD spectra and **c)** g_{CD} values for the same samples as shown in a. **d)** Absolute values of g_{CD} at 514 nm as indicated in c with a dashed line.

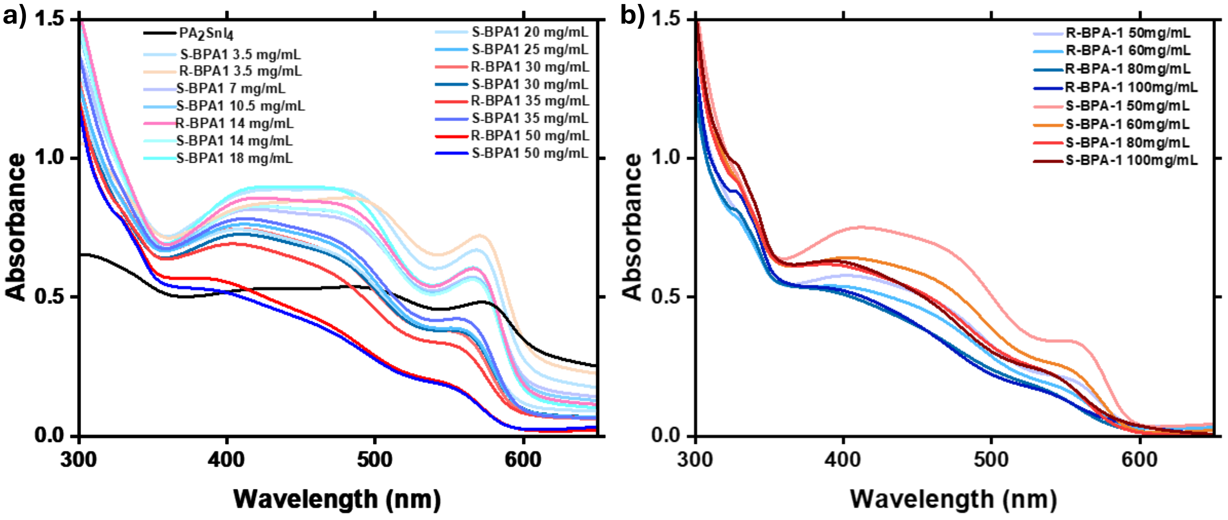

Figure S35: a) UV-Vis of samples containing *R/S*-BPA1 from 3.5 mg/mL to 50 mg/mL, **b)** and until 100 mg/mL

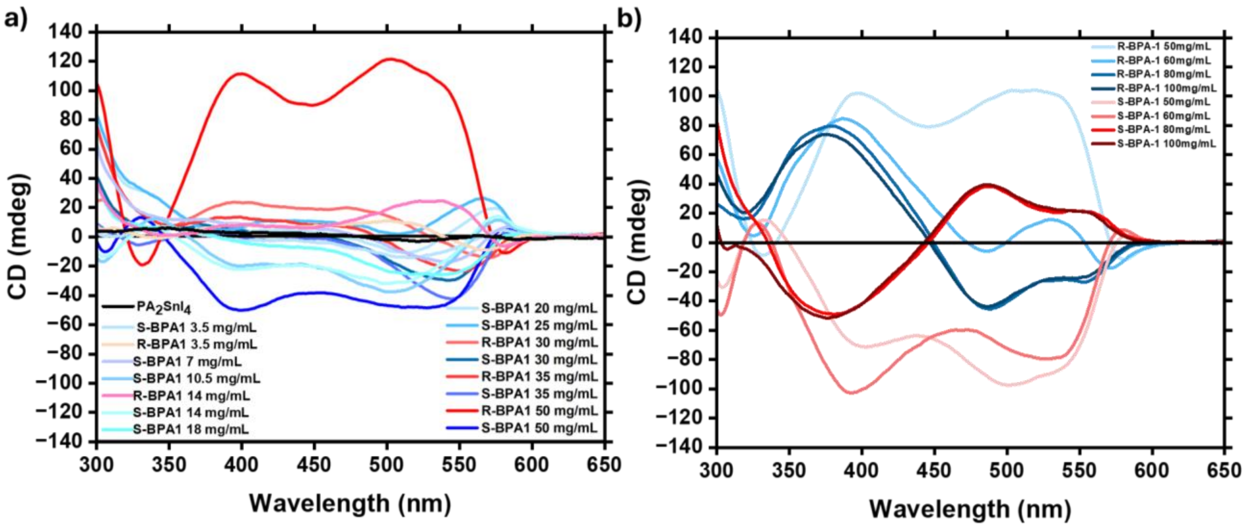

Figure S36: a) CD spectra of samples containing *R/S*-BPA1 from 3.5 mg/mL to 50 mg/mL, **b)** and until 100 mg/mL

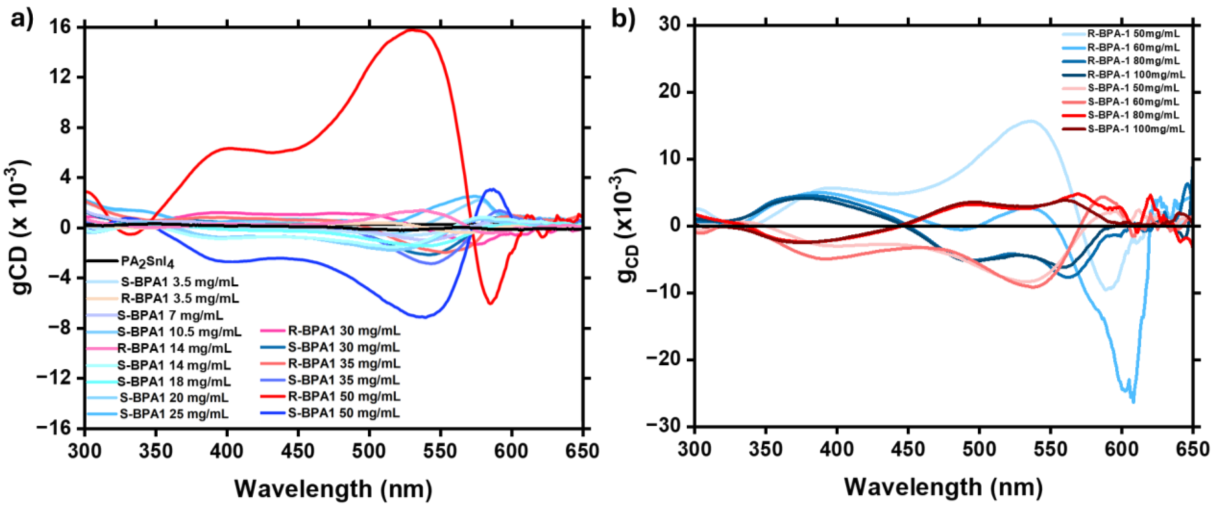

Figure S37: a) g_{CD} spectra of samples containing *R/S*-BPA1 from 3.5 mg/mL to 50 mg/mL, **b)** and until 100 mg/mL.

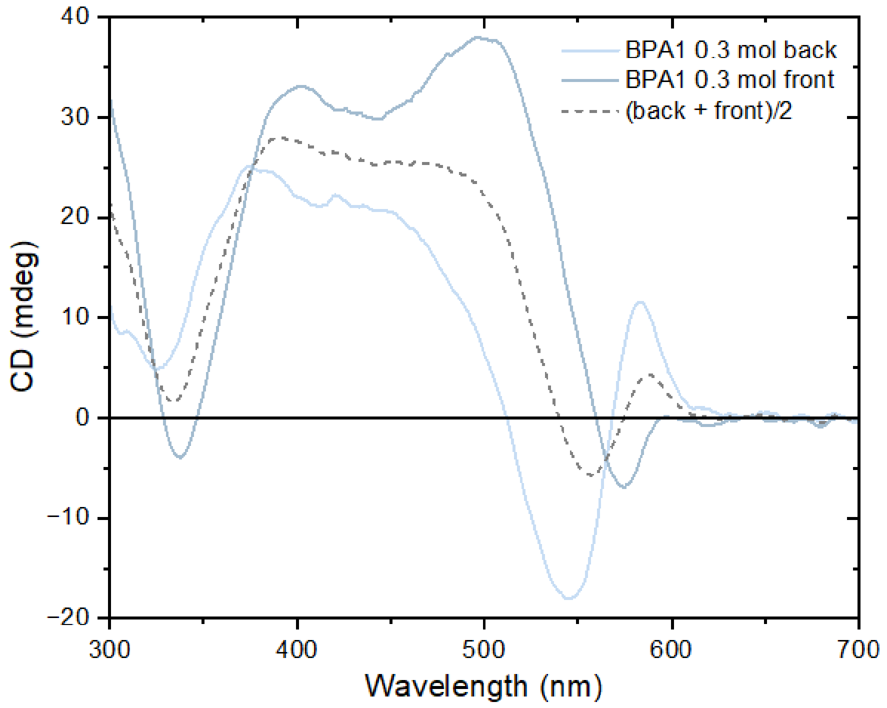

Figure S38: CD spectra of PA_2SnI_4/BPA1 films with 0.3 molar equivalent of BPA1 detected from front and back side of the film. The average curve is dashed.

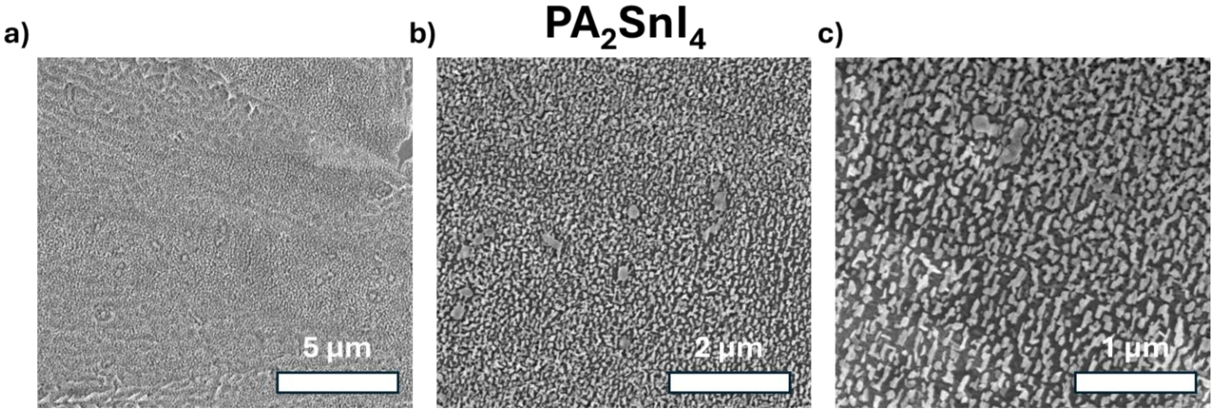

Figure S39: a-c) SEM images of the PA_2SnI_4 control film at 3 different length scales.

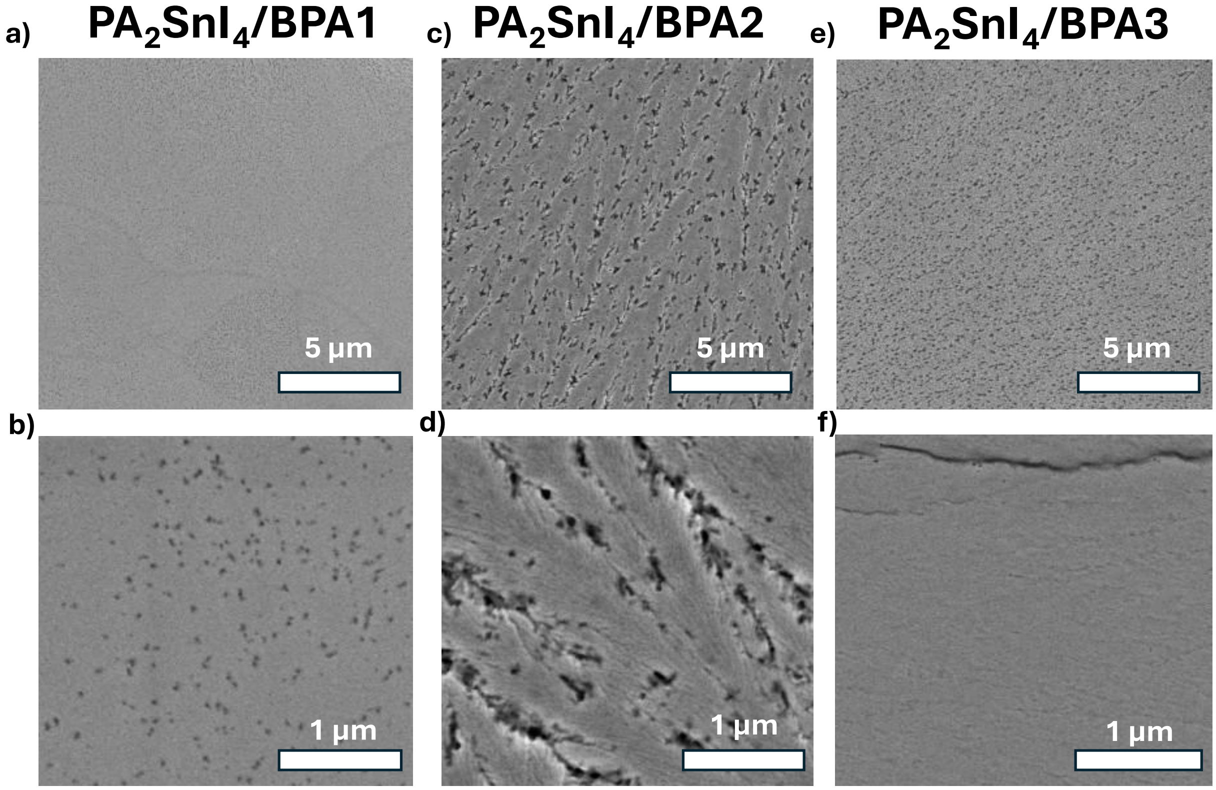

Figure S40: a,b) SEM images of the PA_2SnI_4 control film with the addition of 0.3 molar equivalents of BPA1, **c,d)** BPA2 and **e,f)** BPA3.

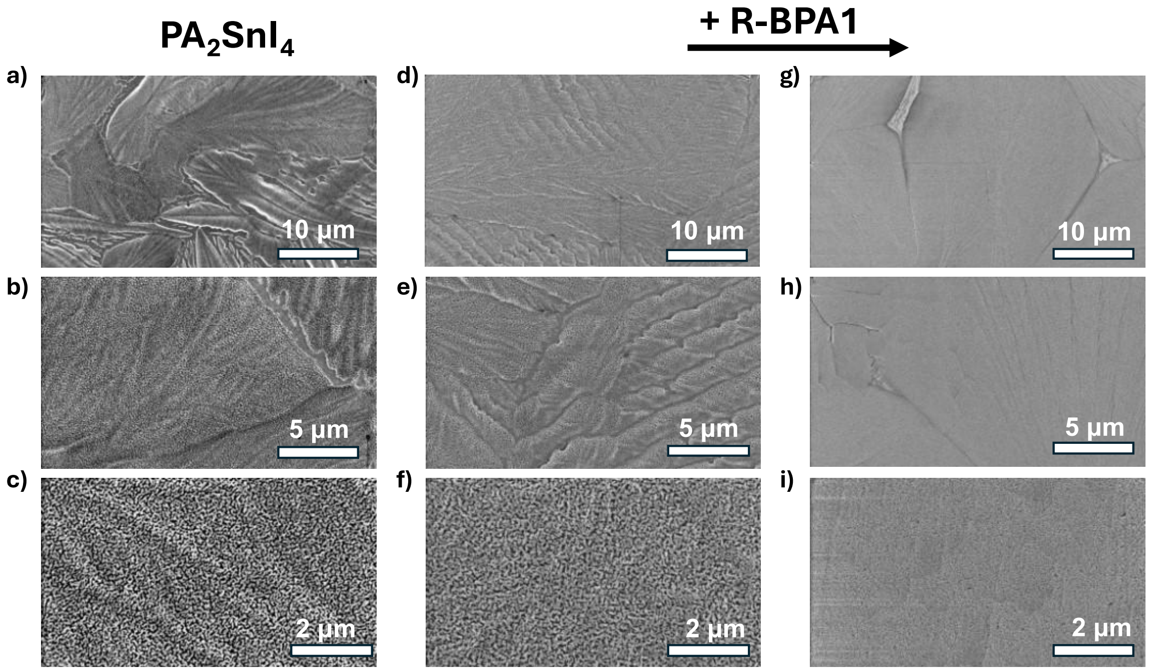

Figure S41: SEM images of PA_2SnI_4 film **(a-c)**, after the addition of 6.6 mg/mL **(d-f)** and 13.3 mg/mL **(g-i)** of BPA1.

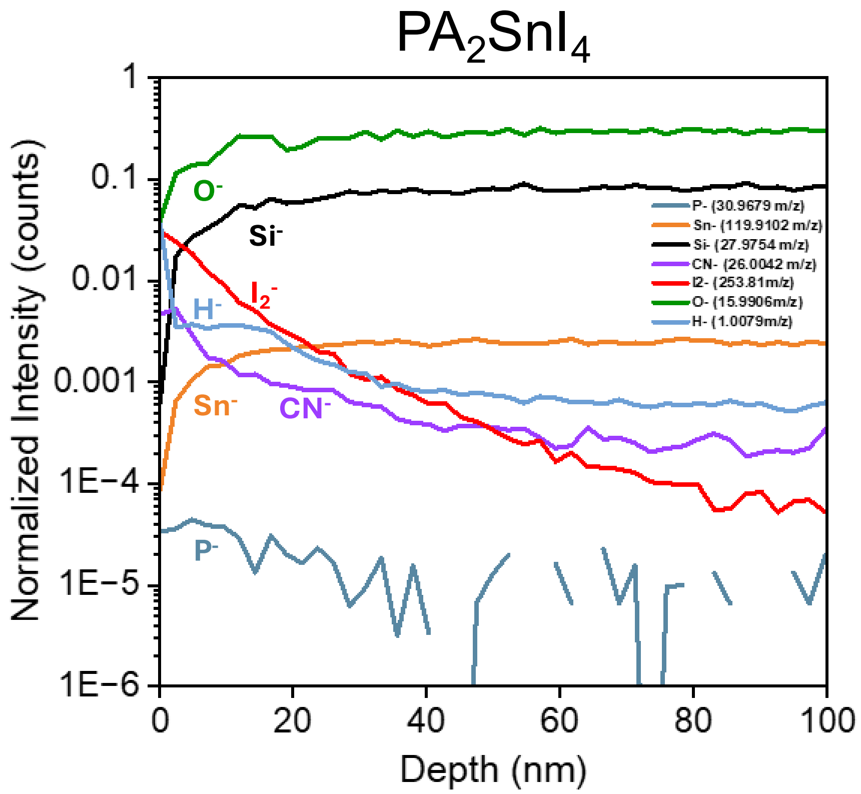

Figure S42: ToF-SIMS depth profiling of the control PA_2SnI_4 film.

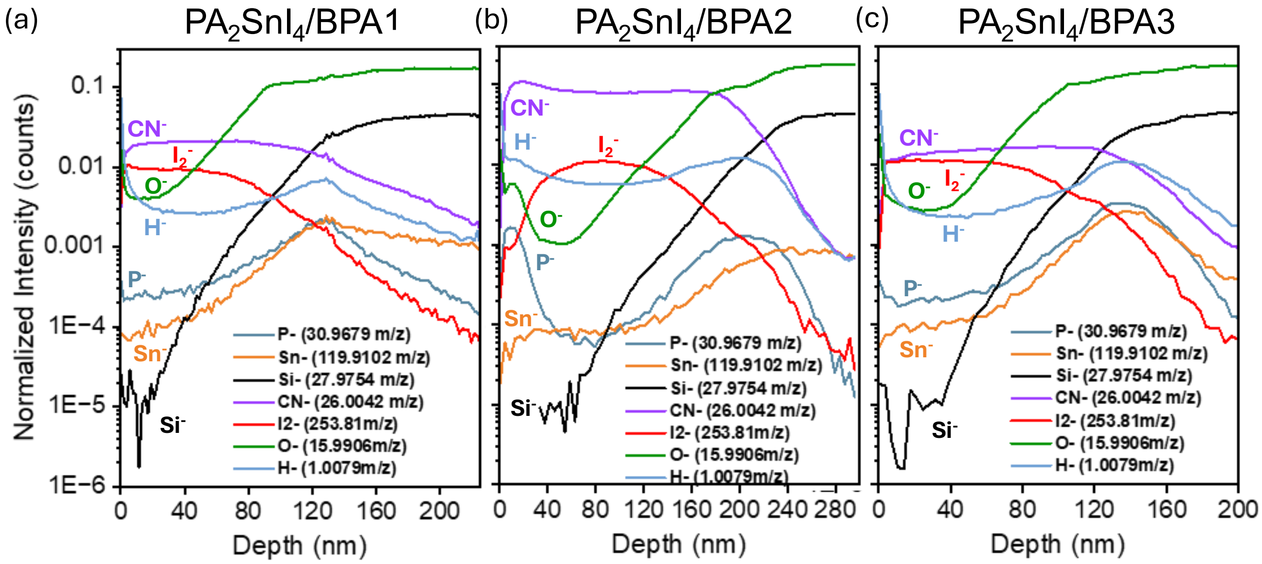

Figure S43: a-c) ToF-SIMS depth profiling of the films with BPA1, 2 and 3.

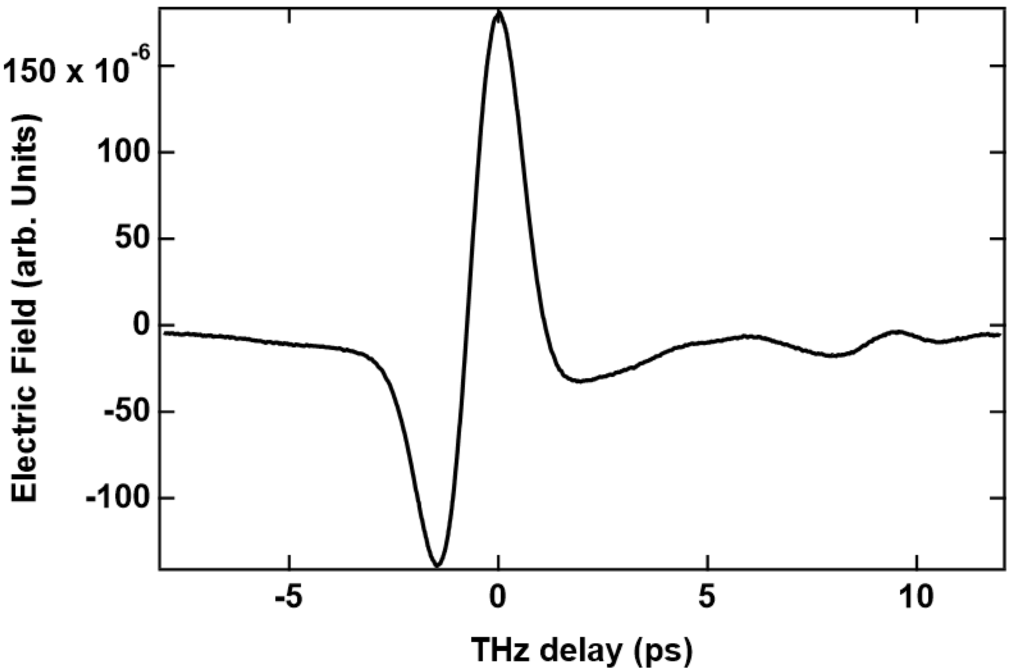

Figure S44: THz electric field in the dark for PA_2SnI_4 control sample.

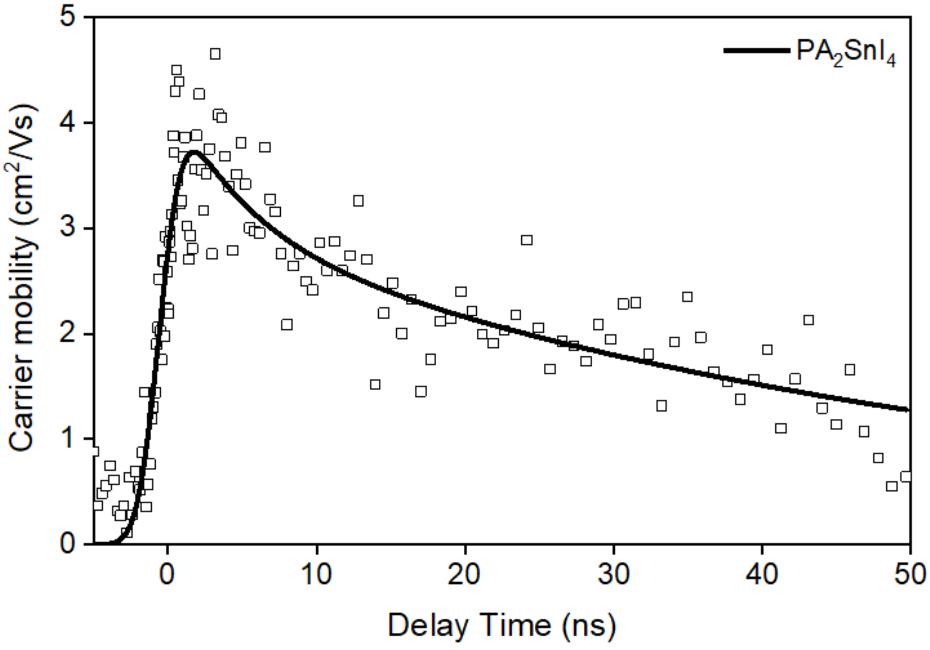

Figure S45: Optical pump terahertz probe on the PA_2SnI_4 control sample with pump fluence of 7 mW/cm^2. The carrier mobility value for the control sample was measured to be 3.72 ± 0.16 cm^2/Vs.

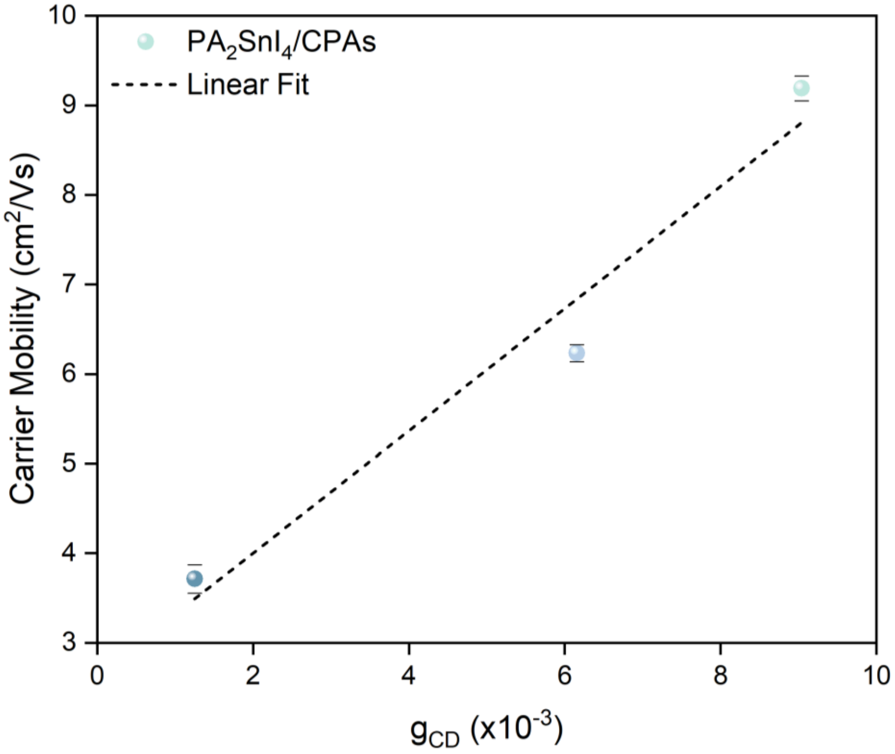

Figure S46: Carrier mobility values from OPTP as a function of g_{CD} values of samples with 0.3 molar equivalents of BPA1 (dark blue), BPA2 (blue) and BPA3 (green). The dashed line indicates the linear fit to the data.

References

(1) Haque, M. A.; Grieder, A.; Harvey, S. P.; Brunecky, R.; Ye, J. Y.; Addison, B.; Zhang, J.; Dong, Y.; Xie, Y.; Hautzinger, M. P.; Walpitage, H. H.; Zhu, K.; Blackburn, J. L.; Vardeny, Z. V.; Mitzi, D. B.; Berry, J. J.; Marder, S. R.; Ping, Y.; Beard, M. C.; Luther, J. M. Remote Chirality Transfer in Low-Dimensional Hybrid Metal Halide Semiconductors. *Nat. Chem.* **2024**, 1–9. https://doi.org/10.1038/s41557-024-01662-2.

(2) Ficks, A.; Sibbald, C.; Ojo, S.; Harrington, R. W.; Clegg, W.; Higham, L. J. Efficient Multigram Syntheses of Air-Stable, Chiral Primary Phosphine Ligand Precursors via Palladium-Catalyzed Phosphonylation of Aryltriflates. *Synthesis* **2012**, *45*, 265–271. https://doi.org/10.1055/s-0032-1316825.

(3) Laue, M.; Schneider, M.; Gebauer, M.; Böhlmann, W.; Gläser, R.; Schneider, C. General, Modular Access toward Immobilized Chiral Phosphoric Acid Catalysts and Their Application in Flow Chemistry. *ACS Catal.* **2024**, *14* (8), 5550–5559. https://doi.org/10.1021/acscatal.4c00985.

(4) Hayasaka, H.; Miyashita, T.; Nakayama, M.; Kuwada, K.; Akagi, K. Dynamic Photoswitching of Helical Inversion in Liquid Crystals Containing Photoresponsive Axially Chiral Dopants. *J. Am. Chem. Soc.* **2012**, *134* (8), 3758–3765. https://doi.org/10.1021/ja2088053.
